# A plant-by-plant strategy for high-ambition coal power phaseout in China

**DOI:** 10.1038/s41467-021-21786-0

**Published:** 2021-03-16

**Authors:** Ryna Yiyun Cui, Nathan Hultman, Diyang Cui, Haewon McJeon, Sha Yu, Morgan R. Edwards, Arijit Sen, Kaihui Song, Christina Bowman, Leon Clarke, Junjie Kang, Jiehong Lou, Fuqiang Yang, Jiahai Yuan, Weirong Zhang, Mengye Zhu

**Affiliations:** 1 Center for Global Sustainability, School of Public Policy, University of Maryland, College Park, MD USA; 2 Department of Geographical Sciences, University of Maryland, College Park, MD USA; 3 Joint Global Change Research Institute, Pacific Northwest National Laboratory, College Park, MD USA; 4 La Follette School of Public Affairs, University of Wisconsin-Madison, Madison, WI USA; 5 Natural Resources Defense Council, Beijing, China; 6 School of Economics and Management, North China Electric Power University, Beijing, China; 7 School of Public Policy and Management, Tsinghua University, Beijing, China

**Keywords:** Climate-change mitigation, Climate-change policy, Energy policy, Environmental impact

## Abstract

More than half of current coal power capacity is in China. A key strategy for meeting China’s 2060 carbon neutrality goal and the global 1.5 °C climate goal is to rapidly shift away from unabated coal use. Here we detail how to structure a high-ambition coal phaseout in China while balancing multiple national needs. We evaluate the 1037 currently operating coal plants based on comprehensive technical, economic and environmental criteria and develop a metric for prioritizing plants for early retirement. We find that 18% of plants consistently score poorly across all three criteria and are thus low-hanging fruits for rapid retirement. We develop plant-by-plant phaseout strategies for each province by combining our retirement algorithm with an integrated assessment model. With rapid retirement of the low-hanging fruits, other existing plants can operate with a 20- or 30-year minimum lifetime and gradually reduced utilization to achieve the 1.5 °C or well-below 2 °C climate goals, respectively, with complete phaseout by 2045 and 2055.

## Abstract (web summary)

A key strategy for meeting China’s 2060 carbon neutrality goal and the global 1.5 °C climate goal is to rapidly shift away from unabated coal use. Here, the authors detail how to structure a high-ambition, plant-by-plant coal phaseout in China while balancing multiple national needs.

## Introduction

An important near-term strategy to address global climate change is to rapidly phase out the use of coal in the global energy system^1^. This includes that existing coal-fired power plants retire at a faster pace, which will be further accelerated if new projects at the planning stage continue to be built^2^. China recently announced the climate goal to achieve carbon neutrality before 2060. While having in place the world’s largest and still growing coal power infrastructure, China faces great challenges to accomplish a rapid coal phaseout in the next few decades towards net-zero emissions.

China’s total installed coal capacity, estimated at 1050 GW, is larger than all other countries’ combined^3^. Moreover, Chinese coal fleet is younger than the global average. The majority of existing coal plants in China have operated less than 15 years and thus have longer remaining lifetimes when compared with the older infrastructure in places like the United States or European Union^3^. As a result, accelerated retirement of coal plants creates higher risks of asset stranding in China. Potential continued coal expansion in the near-term further exacerbates the lock-in effect, which leads to larger economic impacts^4^.

The overwhelming magnitude of existing coal infrastructure makes it highly uncertain if China can decarbonize its heavily coal-reliant power system. While several studies have quantified emission reduction and technology transition pathways in the Chinese power system under different climate goals, they look at coal phaseout in aggregate capacity or generation terms^4–8^. Other global and regional studies have used plant-level data to improve estimates of committed emissions and fossil fuel phaseout pathways^9–13^ as well as stranded assets^14^. Other analyses provide insights on the large variation among individual coal plants, for example by identifying super-emitters^15^ and evaluating the profitability of individual plants^16,17^. However, none of the plant-specific metrics has been assessed for prioritizing retirement; and there is limited understanding about the implications for individual coal plants under rapid energy transition^2^.

In this research, we detail how to structure a high-ambition coal phaseout in China by combining plant-level data, multiple retirement criteria, and long-term scenario analysis through a state-of-the-art global integrated assessment model. Specifically, we answer the questions: When balancing multiple technical, economic, and environmental criteria, which plants can retire first, and which can retire later? What retirement schedules for individual coal plants are compatible with the 1.5 °C and 2 °C climate goals, and what are the implications for operational lifetimes and utilization?

We first simulate mitigation pathways for the global 1.5 °C and 2 °C goals using the China-focused version of Global Change Analysis Model (GCAM-China) and find that China achieves net-zero carbon emissions by 2055 and 2070, respectively, in these scenarios; and unabated conventional coal power generation peaks in 2020 and is phased out by 2045 and 2055, respectively. We then evaluate the 1037 coal-fired power plants currently operating in China according to a total of eight different technical, economic, and environmental criteria and find that 18% of them perform poorly across all criteria evaluated, and that these low-hanging fruits can be retired first and rapidly. We then use the combined retirement metric to rank individual coal plants in the order of retirement priority and combine this analysis with our coal power generation pathways to identify plant-by-plant coal phaseout strategies. Compared to a design where plants maintain today’s operating hours but shut down more rapidly in the near term, gradually reducing plant utilization can guarantee the majority of existing plants a minimum operational lifetime of 20 to 30 years. These findings can help inform the power sector pathways towards China’s 2060 carbon neutrality goal.

## Results

### Coal generation pathway under 1.5 °C

China’s decarbonization pathways suggest that any addition of new coal plants is not in line with the Paris climate goals. Here, we use a global integrated assessment model that has subnational details of China (GCAM-China)^18^ to explore power generation pathways through 2100 that are consistent with the 1.5 °C and 2 °C temperature goals (see Model and Scenarios in Methods).

In our scenarios, China’s net CO_2_ emissions peak around 11 Gt around 2020 and then reach zero by 2055 under 1.5 °C and by 2070 under 2 °C (see Methods). The emission reductions are associated with rapid shift away from coal to low carbon technologies. China’s electricity generation from conventional coal-fired power plants without carbon capture and storage (CCS) also peaks in 2020 and then continues to decline by more than 90% in 2040 and 2050 and drops to zero by 2045 and 2055 under the 1.5 °C and 2 °C targets, respectively (see Methods). A wide range of alternative technologies will be deployed to displace conventional coal plants, dominated by solar and other renewables at the national level (see Methods). Across provinces, renewable energy including solar, wind, hydro, and bioenergy will provide 56% (Guangdong) to 95% (Qinghai) of total electricity generation by 2050 under 1.5 °C, while nuclear and fossil energy with CCS make up 5 to 44% (Supplementary Fig. 5).

Continued coal expansion in the near term would largely increase the risk of stranded assets with the long-term climate goals^4,19^. However, we also recognize that in China, a total of 100 GW of coal plants are currently under construction and 106 GW planned^3^, in addition to the 160 GW of projects that have been suspended by the central government through a series of policies since 2016^20,21^. Building new coal plants would shorten the lifetimes of all coal units—at the global level, by five years when completing projects under construction and by 10 years when completing projects that are planned or under construction^2^.

In this paper, we only look at the pathways without any new builds of coal-fired power plants from today to achieve the 1.5 °C and 2 °C targets. In other words, we focus on how to structure a feasible phaseout of all existing coal plants, while acknowledging that the retirement pathways explored fully depend on an immediate halt of new constructions of coal power plants in China.

### Prioritizing retirement of existing coal plants

**Fig. 1:**
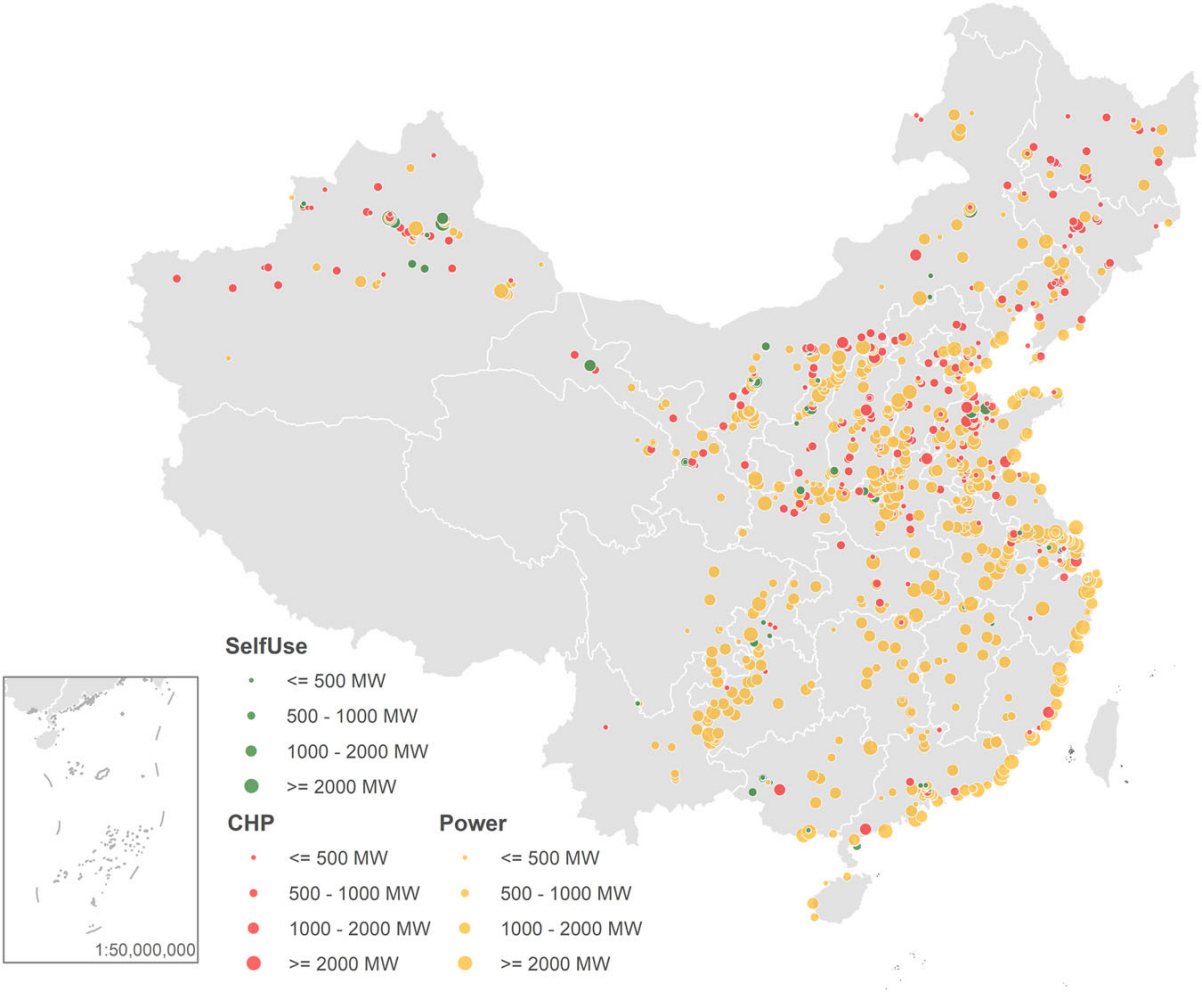
Location, size, and application of existing coal plants in China. Our data covers a total of 1037 coal plants, nearly 3000 individual units (See Data in Methods). Plant size shows the sum of total capacity of all units. Application categorizes coal plants into three types: industrial self-use (captive) plants (SelfUse in green), combined heat and power plants (CHP in red), and power only plants (Power in yellow).

Our data covers a total of 1037 coal plants, nearly 3000 individual units, operating in China (Fig. 1). One plant on average includes about three units, but the actual number may range from one to 12. Our analysis is conducted at the unit level, but to reduce confusion in reading, we refer each unit as a coal plant in the paper, unless it is clearly stated otherwise.

**Fig. 2:**
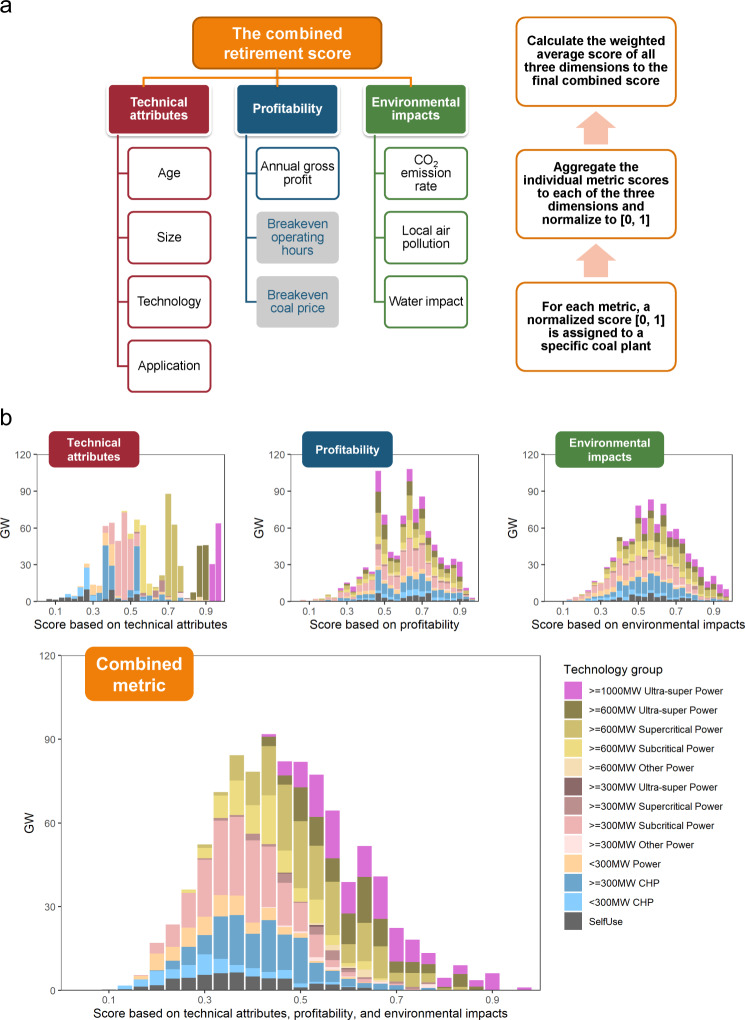
Retirement score of individual coal plants. **a** Methodology of calculating the combined plant-by-plant retirement algorithm: the score of technical attributes is based on the equal-weighted average of plant age, size, combustion technology, and application; the score of profitability is based on capacity weighted annual gross profits; and the score of environmental impacts is based on the equal-weighted average of CO_2_ emission rates, air pollution and health impacts, and water impacts. **b** The combined score is based on the equal-weighted average of the three dimension scores (each score is normalized between zero and one). The combined score, from zero to one, ranks all operating plants from first to last for retirement. Overall, plants to retire first are older, smaller, less efficient, self-use plants located in highly air polluted and water scarce regions.

To strategize the phaseout of all the coal plants, we first conduct a systematic evaluation. A three-step retirement algorithm is developed that ranks all plants based on their technical attributes, profitability, and environmental impacts (Fig. 2a). Each of the three dimensions is quantified through a set of criteria, including plant age, size, combustion technology, application, annual gross profit, carbon dioxide (CO_2_) emission rate, local air pollution, and water impact. We first rate all the plants (with a normalized score from zero to one) for each of the eight criteria. We then aggregate these scores up to each of the three dimensions (again with a normalized score from zero to one). Last, we calculate a weighted average score of all three dimensions to yield the combined retirement score for each plant (see Retirement Algorithm in Methods).

Our core scenario applies an equal weighting at each of the two aggregation stages – first within each dimension and then across the three dimensions. This weighting method is illustrative, and actual retirement pathways would be determined by a range of policy and regional priorities. We perform a sensitivity analysis of the retirement algorithm design (see Sensitivity in Methods and Supplementary Information) and find that while individual plants can be affected by the choice of algorithm, regional pathways are fairly robust to the choice of algorithm.

The retirement priority of all plants is ranked according to the combined score between zero and one. A score close to zero indicates a plant has a lower ranking in the average performance across all three dimensions and thus should retire first; a score close to one indicates the plant has a better performance and will be retiring later in the queue.

According to the retirement algorithm, large and more efficient power plants as well as large combined heat and power (CHP) plants receive a higher technical score, while older, smaller, less efficient plants, and industrial captive plants receive a lower technical score (Fig. 2b). Meanwhile, distribution of neither profitability nor environmental scores shows a clear pattern across different technical groups of plants. In other words, our method shows a plant’s profitability and environmental impacts are not closely related to its technical attributes. This is because several criteria have stronger regional effects (Supplementary Fig. 8). For example, local air pollution and human health impact is assessed by looking at the population weighted PM_2.5_ concentration level at a plant’s location (Supplementary Fig. 4a). It indicates the potential health benefit obtained by closing coal plant in that gridded cell, assuming the same level of air pollution control implemented (see Supplementary Methods for the calculation of individual criteria).

In general, the combined score shows that plants that receive a lower score and first to retire are generally older, smaller, less efficient, and industrial self-use plants (Fig. 2b), and are more likely to locate in highly air polluted, population-dense, and water-scarce regions. While balancing different priorities, all of the most efficient ultra-supercritical plants (600 MW and 1000 MW) receive an above-average score and will be the last to retire.

Across provinces, coal plants in Shanghai, Shandong, Heilongjiang, Hebei, Gansu, Liaoning, Shanxi, Jilin, Qinghai, and Henan receive the lowest scores on average (Supplementary Fig. 8). As a result, these provinces tend to have a faster coal retirement rate than others. This is driven by different factors across provinces. Some are mainly due to undesirable technical attributes, like the aging plants located in Shanghai and the three provinces – Heilongjiang, Liaoning, and Jilin – in the northeast of China. Others are driven by the large health and water impacts, such as those in Shanghai, Shandong, Hebei, and Qinghai, and others are mainly because of low profitability, such as plants in Gansu.

**Fig. 3:**
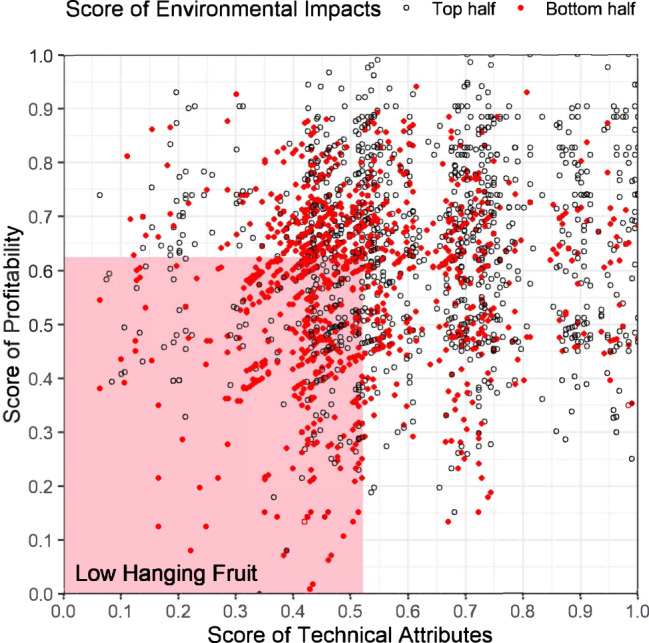
Scores of technical attributes, profitability, and environmental impacts for the low-hanging fruit plants. These plants belong to the bottom 50% for each of all three dimensions evaluated, which is indicated by the red dots (bottom half plants of the environmental score) within the bottom-left pink area (bottom half plants of the technical and profitability scores).

Moreover, we identify 18% of all plants, or a total of 111 GW of capacity (11%), as particularly suitable for rapid near-term retirement—the low-hanging fruit plants. They belong to the bottom 50% for each of all three dimensions evaluated (Fig. 3), and therefore can retire first regardless of which criteria are prioritized in the decision-making. These plants often have been operating for more than 10 years, have a smaller unit size below 600 MW, and use the less efficient subcritical combustion technologies. About 23% of all self-use plants are identified as low-hanging fruits, higher than the rates of power only (19%) and CHP plants (17%).

These low-hanging fruit plants are concentrated in the northeast and central east of China. Specifically, 60% of them, a total of 68 GW, are located in six provinces, including Shandong, Inner Mongolia, Henan, Hebei, Jiangsu, and Shanxi. Moreover, in provinces like Hebei, Heilongjiang, Shanghai, and Shandong, more than 20% of existing coal capacity is identified as low-hanging fruit plants (Supplementary Table 5). Rapid shutdown of these plants is manageable given its relatively small share of the provincial total.

### Developing the plant-by-plant retirement strategies

Combining the top-down national coal power generation pathways from integrated assessment modeling and the bottom-up plant-by-plant retirement priority, we then design two alternative phaseout strategies in support of the 1.5 °C and 2 °C goals. The first scenario, ***constant utilization***, assumes all coal plants will continue to operate at today’s utilization level until they are retired. The retirement schedule will follow the plant-by-plant retirement algorithm starting from the lowest to highest of the combined score. The second scenario, ***guaranteed lifetime***, assumes a policy regime in which most existing coal plants—except for the low-hanging fruit—are allowed to operate through a minimum lifetime, specifically, of 30 years under the 2 °C scenario and 20 years under the 1.5 °C scenario.

Under both scenarios, the same coal power generation constraints are met; however, reduction in coal capacity varies, particularly in the near term. Moreover, the two strategies have different implications on individual coal plants’ operational lifetimes and utilization levels.

**Fig. 4:**
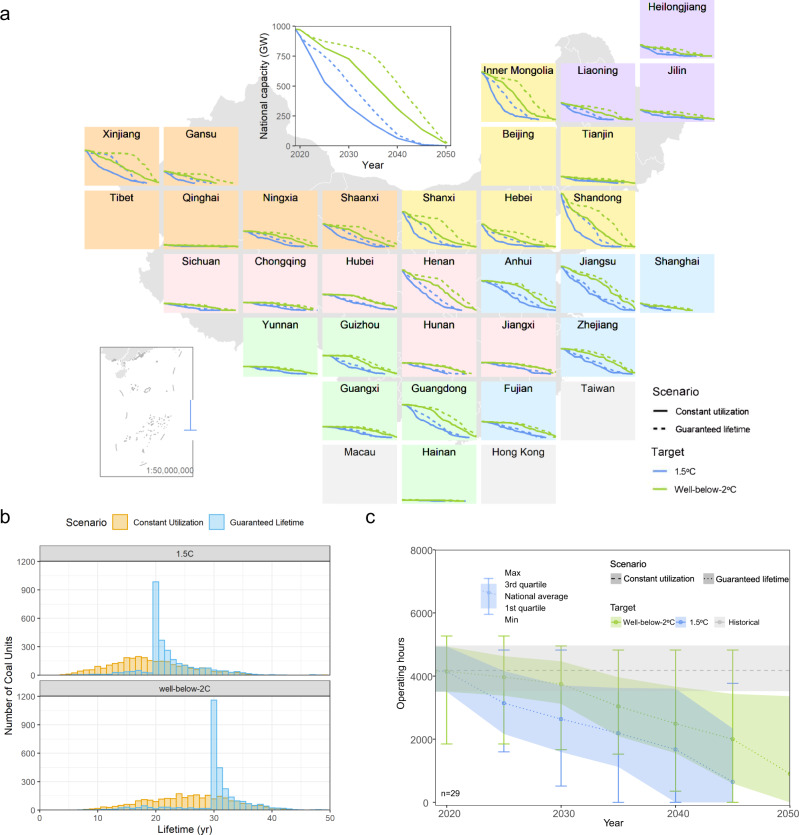
National and provincial coal phaseout pathways and implications on coal plants’ operational lifetimes and utilization levels under two different strategies. **a** Coal plants retirement pathways under the constant utilization scenario (solid line) and the guaranteed lifetime scenario (dashed line), under 1.5 °C (blue) and 2 °C (green), respectively. Compared to the constant utilization scenario, national retirement pathways are delayed by about 5 years the guaranteed lifetime scenario; regional impact varies largely across provinces. **b** Distribution of operational lifetimes of coal plants under the constant utilization scenario (yellow bars) and the guaranteed lifetime scenario (blue bars), under 1.5 °C (top panel) and 2 °C (bottom panel), respectively. **c** Range of plant operating hours under the constant utilization scenario (gray area) under both climate targets, and the guaranteed lifetime scenario under 1.5 °C (blue area) and 2 °C (green area), respectively. The bars indicate the full range of minimum to maximum values across provinces, and the areas indicate the first to third quartile. National average is indicated by the lines. Due to the delayed retirement of coal plants, remaining capacity must operate at gradually reduced hours.

First, we develop the national and provincial retirement pathways. At the national level, retirement of coal plants with guaranteed lifetime is delayed by about 5 years during the next one or two decades until almost the phaseout year—2040 and 2050, under 1.5 °C and 2 °C, respectively. However, the regional impact can be highly variable. About half of the provinces including Jiangsu and Zhejiang show less than three years’ difference in terms of the two retirement pathways, while a few other provinces including Xinjiang, Shanxi, Shandong, Shaanxi, and Gansu show about 10 years of delay in the retirement pathway with the guaranteed lifetime (Fig. 4a). This is because some of the newest plants are retired based on other non-age-related criteria under the constant utilization scenario. These are, for instance, industrial self-use plants in Xinjiang, unprofitable plants in Gansu and Shanxi, plants located in highly polluted and populated area in Shandong. With the guaranteed lifetime, plant age plays a larger role in determining the retirement order, and these plants can get a longer lifetime extension than others.

Second, we look at the implications for plant lifetimes. With constant utilization, the median operational lifetime is 18 and 26 years under the 1.5 °C and 2 °C goals, respectively, while 82% (excluding the 18% low-hanging fruit plants) will operate at least for 20 and 30 years with guaranteed lifetimes under each target (Fig. 4b). These lifetime thresholds are in fact compatible with policy and financial contexts in China. Historically, the average lifetime of retired Chinese coal plants is about 24 years, much shorter than the global average^2^. This is mainly driven by policy efforts to shut down small, dirty units to improve local air quality^22^. Existing experience demonstrates the feasibility of early retirement in this timeframe. Moreover, Chinese coal power plants typically have a 30-year designed lifetime of operation and a 20-year depreciation period^23^. Reaching the designed lifetime or at least the financial depreciation time can help alleviate the immediate financial burden to the project developers and investors.

Third, we assess the implications for plant utilization. The trade-off for the guaranteed lifetime and corresponding delayed retirement of coal plants is that the coal plants must operate at gradually reduced operating hours. In other words, more coal plants with lower utilization generate the same amount of electricity. Under the 2 °C scenario, operating hours on average will be reduced from today’s 4350 h to 3750 h in 2030, 2500 h in 2040, and below 1000 h in 2050. Under the 1.5 °C scenario, it will be reduced to 2640, 1680, and zero hours in 2030, 2040, and 2045, respectively (Fig. 4c). Moreover, across all provinces, coal plants located in about half of the country will be operating below 2000 h by 2040 under 1.5 °C. It indicates that by then a large portion of coal plants will be used for load-following and peaking service only, which would require additional investment to retrofit these plants for higher flexibility.

## Discussion

In this paper, we detail how to structure a plant-by-plant retirement pathway for all existing Chinese coal-fired power plants in support of the global 1.5 °C climate goal, while balancing multiple important national priorities. By combining the top-down long-term scenario analysis from integrated assessment modeling and the bottom-up assessment of more than 1000 operating coal plants in China, we develop the retirement pathways based on global 1.5 °C (and 2 °C) scenarios, retirement priority of individual plants, and specific policy designs for an appropriately paced coal phaseout strategy in China. These pathways are highly relevant to China’s most recent climate pledge to achieve carbon neutrality before 2060, where net CO_2_ emissions reach zero by 2055 (or 2070) under the 1.5 °C (or 2 °C) scenario.

Three key elements are featured in the proposed strategy. First, successful implementation of the pathways depends on an immediate halt of new construction of conventional coal plants in China. The sooner new construction stops, the lower the cost of deep decarbonization will be in the future. Cancellation of planned projects can reduce the risk of stranded assets and enhance the feasibility of existing plants’ phaseout. Second, a small set of existing plants (18%) is eligible for rapid shutdown in the near term, since they perform poorly across all the technical, economic, and environmental criteria assessed. Third, remaining plants can operate through a minimum guaranteed lifetime of 20 (or 30) years, but with gradually and responsibly reduced hours mainly for meeting peak load demand in China’s power system.

The three-part strategy is compatible with an accelerated coal phaseout in 2045 (or 2055) under the 1.5 °C (or 2 °C) climate goal. It also suggests a possible range of coal phaseout pathways in the power sector to achieve China’s 2060 carbon neutrality goal: without new builds, majority of existing coal plants can operate over a minimum lifetime between 20 and 30 years to achieve a phaseout of unabated coal power generation around 2050. However, such a pathway highly depends on taking immediate actions of the “no new coal” strategy, where continued coal builds will accelerate the retirement of all plants and the phaseout timeline.

The retirement algorithm is flexible and can be adjusted to reflect different priorities in local contexts. For example, certain CHP plants, especially those that provide residential heating services, are in fact very critical and not easy to replace in the short term. Meanwhile, some other CHP plants are inefficient options for heating services (for example, for industrial processes) and have already been targeted by policy for early phaseout^24^. To further differentiate CHP plants, we thus test another scoring method that assigns a higher score to those in the northern provinces, potentially used for residential heating. However, we note that accounting for this has little impact on the overall provincial phaseout pathways. Similar conclusions are achieved when testing alternative weighting options for combining metrics into the final retirement score (see Sensitivity in Methods).

In addition to the three dimensions of the retirement algorithm, grid stability and equity are equally, if not more, important in the discussion of coal power phaseout. Together, the five dimensions would be useful to integrate existing and future research from each field into a comprehensive analytical and policy framework.

An accelerated coal phaseout will be accompanied with increasing electricity generation from intermittent wind and solar power. Accommodating high penetration of intermittent electricity from wind and solar is not a unique challenge to China and would require substantial grid management and forward planning. Studies consistently show contributions of well over 50% of generation are viable but will require substantial evolution of grid management approaches^25,26^. This includes some amount of reliable generation capacity to complement intermittent sources. It also includes modernizing grid transmission and distribution, developing next-generation storage and other flexibility technologies, and deploying demand-side management technologies. A deeper assessment of this question is essential to a successful coal transition in China and needs to be answered by future research.

Finally, a successful coal phaseout needs to be equitable. It is also referred as a just transition^27–29^, where potential financial losses, and economic and social impacts, are well managed during the transition. Not only the overall magnitude, but also the distribution of these potential impacts across different regions, different stakeholders, and different demographic groups needs to be evaluated. Moreover, new research should focus on such impacts through the entire supply chain, while at a finer resolution. For example, although employment at coal power plants is only a marginal share of total population (less than 0.1% in most Chinese provinces, Supplementary Fig. 10), the number of employments in coal mining are much larger, and especially in certain regions where local economies and communities are heavily centered on coal. To answer those questions, more research is needed to integrate more research from other disciplinaries (i.e., social behavior, economics) into the coal phaseout strategy in China.

## Methods

### Global Change Analysis Model (GCAM-China)

The Global Change Analysis Model (GCAM, jgcri.github.io/gcam-doc/) is an integrated assessment model that represents and links the world economy, energy, agriculture, land-use, water, and climate systems. It is designed to explore interactions between complex systems and gain insights about long-term trends. GCAM has been widely used to produce scenarios for international and national assessments, including the Intergovernmental Panel on Climate Change (IPCC) report^1,30–32^, the Representative Concentration Pathways (RCPs)^33^, and the Shared Socioeconomic Pathways (SSPs)^34^.

Specifically, GCAM takes in assumptions about population growth and changes in labor productivity, along with representations of resources, technologies, and policies, and solves for the equilibrium prices and quantities of various energy, agricultural, and GHG markets in each five-year period from 2010 (the calibration year) to 2100 at different spatial resolutions. Primary energy (i.e., coal and other fossil fuels), agricultural products, and biomass are traded globally. GCAM tracks emissions of sixteen GHGs, aerosols, and short-lived species endogenously based on the resulting energy, agriculture, and land systems activity. Emissions are then passed to the climate carbon-cycle module and converted to concentrations, radiative forcing, temperature, and other responses to the climate system^35^.

Here, we use a special version of GCAM that provides sub-national ﻿details in the energy markets for China. Specifically, in GCAM-China, the world economy and energy systems operate across 31 geo-political regions plus the further disaggregated 31 province-level sub-regions of mainland China^18^. Population and GDP, as well as energy demand, supply, and transformation are modeled at the provincial level.

Electricity demand of each province is driven by increased electrification in buildings, industrial, and transportation sectors. Each province also has its own electricity supply system. The electricity sector includes a detailed representation of different power generation technologies, including those fueled by coal and other fossil fuels (with and without CCS), bioenergy (with and without CCS), nuclear, and renewables. Cost data of different technologies is based on NREL Annual Technology Baseline data^36^. The availability of wind and solar resources and carbon storage differs by province and is represented by provincial-specific resource curves. The deployment of hydro and nuclear power plants in future is based on the plan of the Chinese government, as these investment decisions are often driven by factors beyond costs^37^. The deployment of other electricity generation technologies depends on relative costs and is achieved using a choice function that is designed to represent decision making among competing options when only some characteristics of the options are observed^38^. For electricity trade between provinces, we group provinces into 6 different grid regions, consistent with power grid regions in China – North, Northeast, East, Central, Northwest, and South. Provinces within the same grid region can trade freely within that region, while trade between grid regions is limited.

Individual coal plants’ shut down schedules are calculated to match the overall national retirement pathway in GCAM-China. GCAM-China balances total electricity demand and supply at the grid region level (Supplementary Fig. 7a). When comparing the coal pathways from GCAM-China (“top-down”) and the plant-by-plant retirement results (“bottom-up”) for each grid region, we find that some regions show more divergence than others, but only two regions have a potential issue that coal plants retire too quickly than the top-down results suggested due to their “worse” performance.

The top-down and bottom-up coal pathways are almost identical for the Central China Grid and China Southern Power Grid (Supplementary Fig. 7b). This indicates that these two grids can follow our plant-by-plant retirement schedule and meet future demand without trading electricity with other regions. In contrast, the bottom-up coal retirement is faster than the top-down phaseout in the North China Grid and Northeast China Grid but slower in the Northwest China Grid and East China Grid (Supplementary Fig. 7b). These differences could be addressed by increased investment in clean energy technologies or through long-distance transmission. For example, the Northwest China Grid and East China Grid could export electricity to other regions, while the North China Grid and Northeast China Grid could import electricity.

### Scenarios

Using GCAM-China, we develop two deep decarbonization scenarios by limiting end-of-century radiative forcing at different levels. Specifically, a well-below 2 °C and a 1.5 °C scenario, has the end-of-century radiative forcing at 2.6 Wm^−2^ and 2.0 Wm^−2^, respectively. Starting in the model period of 2025, we apply an increasing global carbon price on fossil fuel energy-related emissions across regions and sectors that is consistent with the well-below 2 °C or 1.5 °C temperature goal. This carbon price is applied to all regions and all sectors of the economy and emission reductions occur where it’s economical. Therefore, our results show how much mitigation would happen in the power sector with all other sectors mitigating at the same marginal abatement costs. The implication of different sectoral policies is an important research topic but beyond the scope of this research. However, we note that the literature generally points to the importance of decarbonizing the electricity sector early and quickly, especially given that mitigation pathways in other sectors frequently involve electrification. Reflecting institutional difficulties associated with pricing carbon in land, only 10% of the carbon price is passed on to the land sector.

We exogenously specify 2010, 2015, and 2020 coal power generation for each vintage group by province to match historical data and our plant-by-plant dataset. We categorize every coal power plant in a given province into seven categories (vintages) depending on when they started operation: 1975 (or before), 1976–1990, 1991–1995, 1996–2000, 2001–2005, 2005–2010, and beyond 2010.

Historical data up to 2010 is calibrated natively in GCAM. We exogenously specify the 2015 generation of older vintages to the historical value. For estimating the 2020 generation for the older vintages, we use the following procedure. First, we assume that the 2018 aggregate coal-fired power plant generation value will hold true for 2020. Second, we subtract the estimated 2020 generation for the “Beyond 2010” vintage to obtain the total aggregated generation for all the older vintages. Third, we assume that the ratio of contribution of each vintage to the aggregate generation will be the same as 2015. Finally, we adjust the s-curve generation between 2015 and 2020 reflecting these assumptions.

For the new “Beyond 2010” vintage, we exogenously specify the 2015 vintage to the historical values, and 2020 vintages to those currently under construction. We assume the 2015 generation values to hold true for 2020 for power plants that were operating between 2011 and 2015. We calculate projected generation from power plants that started operation (or were expected to do so in the case of dates beyond 2018) between 2016 and 2020. We add the two values to obtain an expected “Beyond 2010” vintage 2020 generation value for each province.

For 2020, total coal power generation is matched with 2018 data. Since the model runs at a five-year interval, we use 2018 generation data to approximate the trend between 2015 and 2020. Starting in the next model period of 2025, the model finds the most cost-effective pathways to achieve the 1.5 °C or 2 °C climate targets through a global carbon price. When converting to the coal plants retirement pathways (GW), our baseline value is based on the plant-level data up to May 2019, and we used a linear interpolation between the 2019 data and the first model period in 2025, and between all model periods thereafter, to calculate the annual retirement pathways.

**Fig. 5:**
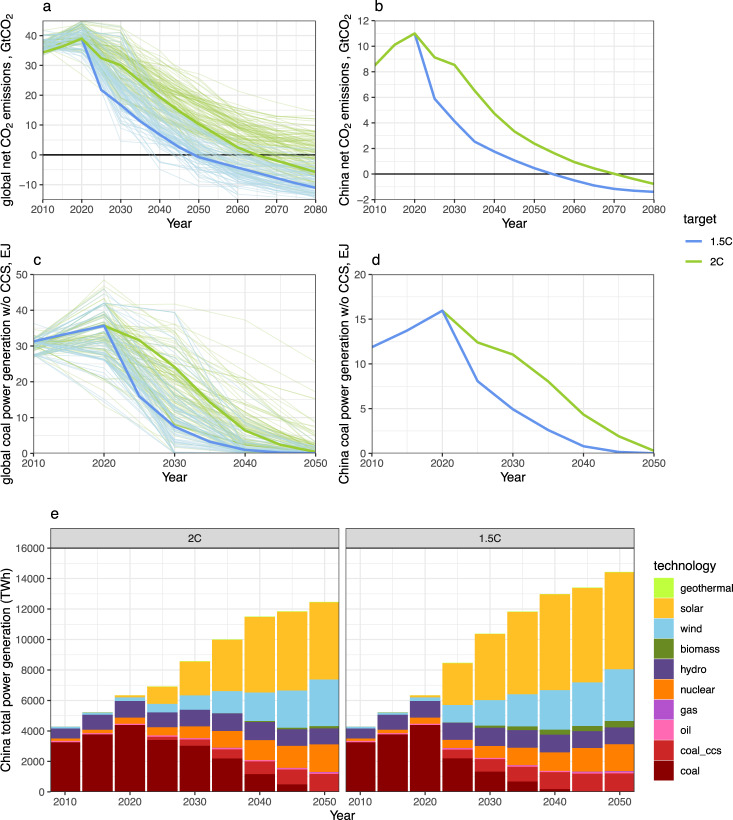
Global and China 1.5 °C and 2 °C scenarios, 2010–2050. **a** Global net CO_2_ emission pathways, **b** China net CO_2_ emission pathways, **c** global conventional coal power generation pathways, **d** China conventional coal power generation pathways, and **e** China electricity generation by technology. The lighter lines are scenarios from the IAMC 1.5 °C Scenario Explorer. The light blue lines indicate scenarios categorized as “Below 1.5 °C”, “1.5 °C low overshoot”, and “1.5 °C high overshoot”; the light green lines indicate scenarios categorized as “Lower 2 °C” and “Higher 2 °C” in the database.

Our scenario shows that both global and China’s net CO_2_ emissions peak around 2020, and then China reaches net-zero emissions by 2055 under 1.5 °C and by 2070 under 2 °C, while the world reaches net-zero carbon five years earlier under each target (Fig. 5a, b). This is associated with global and China conventional coal power generation also peaking in 2020, and being phased out around 2040 and 2050, under 1.5 °C and 2 °C, respectively (Fig. 5c, d). China’s total power generation will increase to about 12,500 TWh under 2 °C and to about 14,500 TWh under 1.5 °C by 2050, mainly supplied by solar and other renewable energies (Fig. 5c).

When comparing the global pathways from our scenarios against the ensembles from the IPCC 1.5 °C database^39^, it shows that our results are within the range of the literature (Fig. 5a, c); however, the 1.5 °C database does not provide regional specific results for China, making a direct comparison more challenging. Nonetheless, looking at the global pathways, although there seems to be large uncertainty on near-term behaviors, models tend to highly agree on the long-term phaseout timeline of conventional coal power generation at the global level – that is, by around 2040 for 1.5 °C and by around 2050 for 2 °C. It suggests that across these scenarios, China’s coal phaseout also needs to happen no later than these timelines, consistent with our results.

Moreover, different technology futures or electricity demand has little impact on coal generation pathways under deep decarbonization scenarios. For example, the comparison with the IPAC results illustrates that although different models have very different projections and interpretations about how China will achieve power system deep decarbonization (i.e., through different combinations of alternative technologies), the retirement pathways of conventional coal power generation to achieve the climate goals are consistent and robust (Supplementary Fig. 6a).

Moreover, low electricity demand only marginally delayed the coal power decline. In addition to the core scenario (low demand) used for plant-by-plant retirement, we also looked at a high energy demand scenario for each climate target. With higher energy demand to achieve the same emission pathways, power generation from conventional coal plants will need to decline faster (Supplementary Fig. 6b, c) to offset the increased emissions in other sectors, mainly buildings and industry (Supplementary Fig. 6d). This indicates that improved efficiency (as in the low demand scenario) can slightly slow down coal phaseout in the near term.

### Data

We employed unit-level data of coal power plants that are operating in China by May 2019 from an existing dataset^40^ with independent modification and updates based on primary research. The dataset covers 1037 operating coal plants, nearly 3000 individual generators, a total of 980 GW. According to China Electricity Council (CEC), the total capacity of coal units is estimated to be 1008 GW by the end of 2018^41^. Our data covers more than 95% of the total capacity list by CEC.

A number of variables, either collected or estimated at the unit level, are used in the calculation of metrics, including location, capacity, vintage year, combustion technology, application, heat rate, coal type, and project developer. To get a more up-to-date version, it is further modified with more recently built power plants during the months of February to May of 2019. The reference data of our update is according to the latest information published on the Beijixing power website^42^, one of the biggest online platforms of the power sector in China, as well as other related documentation from reliable sources. A summary table (Supplementary Table 1) that describes the data in more detail is provided in Supplementary Methods.

### Retirement algorithm

We developed the three-step retirement algorithm based on multi-criteria decision-making (MCDM) method^43,44^. First, we select a set of criteria to evaluate individual coal plants. Specifically, technical attributes refer to individual plants’ engineering features and are described with four metrics: age, size, combustion technology, and application. The profitability of a plant is assessed through an estimate for gross profit, calculated as the difference between annual revenue and annual cost in the current year. Environmental impacts are drawn from three areas: global climate change impact evaluated with a plant’s CO_2_ emission rate, local air quality, and human health impact assessed with the population-weighted PM_2.5_ concentration level of a plant’s location, and water impact estimated with the water risk level of a plant’s location (see Supplementary Methods for the calculation of individual criteria).

In particular, the local air pollution and human health impact metric and the water impact metric characterize the overall environment in which the plant is operating, whereas the technical metrics capture the characteristics of individual plants. Other conditions equal, shutting down a coal plant operating in a highly polluted or water scarce environment leads to larger marginal benefits, and thus such a coal plant has a higher retirement priority than those located in an environment with less pollution and water scarcity. These environmental metrics have limitations in that they do not capture the exact air pollution impact from each coal plant. Our choice to use these metrics is partially related to data limitations on plant-level air pollution control technologies.

We use the environmental metrics to focus on the need for continuous air quality improvement in the long run. In recent years, China’s coal plants have significantly reduced air pollutant emissions through widely installed end-of-pipe emission controls. The implementation rates of SO_2_, NO_x_, and primary PM2.5 control technologies reached approximately 96, 93, and 100%, respectively, in 2018^45^. As a result, end-of-pipe control is less relevant in differentiating the majority of the operating plants for retirement. Moreover, it also has limited potential to continuously improve air quality to the WHO guideline^46^ level of 10 μg/m^3^. Such long-term improvements require a different strategy – that is, an energy system transition from coal to renewables^47^.

To incorporate multiple criteria into the decision process, we start with assigning each plant a normalized score ranging between zero and one for each individual criterion. The normalized scores range from zero to one, which are calculated using Eq. 1 based on the commonly used range normalization approach. A score close to zero indicates the plants are the first to retire; a score close to one indicates they are the last to retire.

1 $${\mathrm{Score}}\;{\mathrm{for}}\;{\mathrm{metric}}\;Xi = (x - x{\mathrm{min}})/(x{\mathrm{max}} - x{\mathrm{min}})$$

Next, we aggregate the metric scores to each of the three dimensions by averaging individual metric scores of the given dimension. The dimension score of technical attributes, for example, is equal to the average of the metric scores of plant age, size, technology, and application and also has a value range from zero to one.

2 $${\mathrm{Dimension}}\;{\mathrm{score}}\;D = \mathop {\sum }\limits_{i = k}^1 {\mathrm{Score}}\;{\mathrm{for}}\;{\mathrm{metric}}\;Xi/k$$

Lastly, we take the weighted mean of the three dimension scores as the combined score. The results presented in the main text are based on the equal weighted mean method to combine the three dimension scores. The retirement order of all plants is ranked according to this combined score. A lower score indicates a plant is ranked lower by the retirement order and should retire first.

3 $${\mathrm{Combined}}\;{\mathrm{score}} = (w_{{\mathrm{tech}}} * D_{{\mathrm{tech}}} + w_{{\mathrm{profit}}} * D_{{\mathrm{profit}}} + w_{{\mathrm{env}}} * D_{{\mathrm{env}}})/\mathop {\sum }\limits_{i = 3}^1 w_i$$

### Sensitivities

Power sector’s contribution to local air pollution tends to vary across regions, which may affect the health benefit gained by shutting down coal plants at different locations, and thus affect the retirement priority ranking. Therefore, we use two alternative metrics for local air pollution in the retirement algorithm as sensitivity analysis: percentage of SO_2_ or NOx emissions from the power sector at the plant’s location (see Supplementary Table 1). We find that provincial coal phaseout pathways are robust across different metrics used (Supplementary Fig. 11a, b). This suggests that although individual plants’ ranking may change with different air pollution metrics, the retirement time schedule consistent with the 1.5 °C or 2 °C climate target is not sensitive to this assumption.

Among all the provinces, Inner Mongolia tends to show a relatively larger change in the near-term pathways (Supplementary Fig. 11a, b). Under both the “power SO_2_” and the “power NOx” methods, coal plants located in Inner Mongolia retire slightly faster than the original “total PM2.5 concentration” approach, indicating that the power sector contributes to a larger share of the local air pollutant emissions in the region.

To investigate the uncertainty existing in our retirement algorithm, we perform a sensitivity analysis on the weighting methods to calculate the combined retirement score. First, we tested an “equal-weight” method. Instead of the two-step weighting method applied in the core analysis, we apply an equal weight for all the individual metrics to calculate the weighted average as the combined score. As a result, technical and environmental dimensions will have a larger weight than profitability due to more metrics included. These two weighting options tend to have marginal impact on the provincial coal retirement pathways (Supplementary Fig. 11c).

Second, we tested a “CHP-prioritized” method. Specifically, CHP plants located in northern provinces are given a higher score when rating application as part of technical criteria (see Supplementary Methods and Supplementary Table 2). Northern provinces here include Hebei, Shanxi, Inner Mongolia, Heilongjiang, Jilin, Liaoning, Shaanxi, Ningxia, Gansu, Qinghai, and Xinjiang. This weighting method highlights the important roles of CHP plants in heat generation in northwest China grid, north China grid, and Northeast China grid. Again, these two weighting options tend to have a marginal impact on the provincial coal retirement pathways (Supplementary Fig. 11d).

Level of retirement agreement between two different weighting methods is quantified using the number of coal power units that are agreed to retire by the given year based on both the methods. The overall retirement agreement level is high between the original weighting method and CHP prioritized method, as well as the equal-weighted method under 1.5 °C and 2 °C (Supplementary Fig. 12). The agreement also increases with time as well as the stringency of targets. However, near-term decision making is critical. In the next decade, about a third or half of the retired coal plants will change when different priorities are adopted.

### Reporting summary

Further information on research design is available in the Nature Research Reporting Summary linked to this article.

## Supplementary information

Supplementary Information

Reporting Summary

## Data Availability

Unit-level data of Chinese coal-fired power plants used in this analysis are from the Global Coal Plant Tracker dataset (Global Energy Monitor, Global Coal Plant Tracker, Jan 2019. Available at https://endcoal.org/global-coal-plant-tracker/). Other data used are cited in Supplementary Table 1.

## Code availability

The Global Change Analysis Model is an open-source integrated assessment model, available at: https://github.com/JGCRI/gcam-core/releases. Data processing is conducted using R 3.6.3. All figures are created using R 3.6.3, and ArcMap 10.7 is used to create the maps in Figs. 1 and 3, and Supplementary Figs. 4, 5, 9, and 11.
