# Supplementary material for: A plant-by-plant strategy for high-ambition coal power phaseout in China
Source: Nat Commun. 2021 Mar 16;12:1468. doi: 10.1038/s41467-021-21786-0 (PMC7966364; doi:10.1038/s41467-021-21786-0)
Supplement: Supplementary file 1 — Supplementary Information [file 41467_2021_21786_MOESM1_ESM.pdf]

A plant-by-plant strategy for high-ambition coal power phaseout in China

Cui et al.

Supplementary Information

Supplementary Method: Data

Supplementary Table 1 Description of data used for the retirement algorithm.

Dimension	Variable	Dataset	Reference	Resolution	Year
Technical Attributes	Age	Global Coal Plant Tracker and authors' own data collection	Global Coal Plant Tracker, Global Energy Monitor, Jan (2019)	Plant level	2019
	Capacity				
	Combustion Technology				
	Application				
Profitability	Electricity Price	Electricity Consumption Data	National Energy Administration of the People's Republic of China (NEA). (2016). National Energy Administration Released 2015 Electricity Consumption Data.	Provincial level	2016
	Operating Hours	Operating Hours of Coal Power Data	China Beijixing News, http://news.bjx.com.cn/html/20190214/962680.shtml	Provincial level	2018
	Coal Price	National Development and Reform Commission (NDRC)	The National Development Reform Commission's Report at the 4th Session of 12th National People's Congress: A Review. China Report, 52(3), 228–234. https://doi.org/10.1177/0009445516650957	Provincial level	2015

Environmental Impacts	Coal Type	Global Coal Plant Tracker data complemented by Aden et al. (2008)	Global Coal Plant Tracker, Global Energy Monitor, Jan (2019) Aden, N. T., Fridley, D. G., & Zheng, N. (2008). Outlook and Challenges for Chinese Coal (No. LBNL-4772E). Lawrence Berkeley National Lab. (LBNL), Berkeley, CA (United States).	Plant level	2019
	Annual PM2.5 Concentration	PM2.5 Hindcast Database for China (PHD)	Xue T, Zheng Y, Tong D, Zheng B, Li X, Zhu T, Zhang Q. (2018). Spatiotemporal continuous estimates of PM2.5.	0.1°	2016
	Population Density	UN WPP-Adjusted Population Density v4.11	NASA Socioeconomic Data and Applications Center (SEDAC): UN WPP-Adjusted Population Density, v4.11 (2015).	30 arc-second	2015
	Water Risk	Aqueduct Water Risk Global Maps 2.1 Data	Gassert, F., M. Luck, M. Landis, P. Reig, and T. Shiao. (2014). Aqueduct Global Maps 2.1: Constructing Decision-Relevant Global Water Risk Indicators. Working Paper. Washington, DC: World Resources Institute. Available online at http://www.wri.org/publication/aqueduct-globalmaps-21-indicators	Polygons	2010
	Annual SO2 and NOx emissions by sector	Multi-resolution emission inventory for China v1.2	http://meicmodel.org/index.html<br><br>Zhang et al., 2009[1]<br>Zheng et al., 2014[2]<br>Li et al., 2014[3]<br>Liu, et al. 2015[4]	0.25°	2016

Supplementary Method: Metrics of the Retirement Algorithm

1. Technical attributes

Technical attributes of individual plants are described using four metrics: age (vintage year), size (capacity), combustion technology and application. The majority of China's existing coal fleets were implemented after 2005 (Supplementary Figure 1). The older fleets built before 2005 are mainly 300 MW or smaller units with subcritical technology. The more recent units are featured as 1000 MW or 600 MW ultra-supercritical power plants or large combined heat and power (CHP) plants.

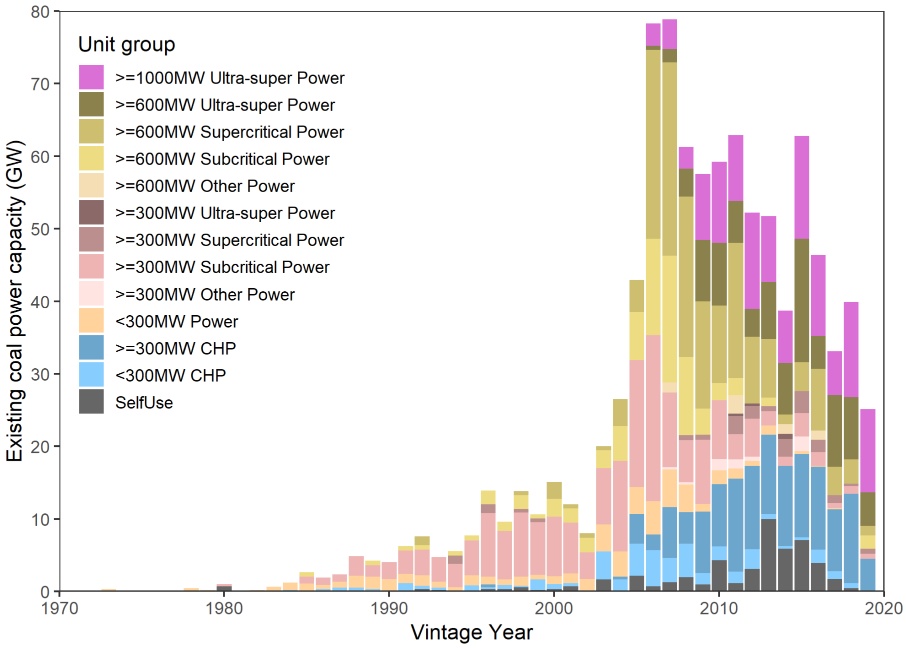

Supplementary Figure 1 Distribution of technical attributes of existing coal plants.

Plant age is quantified using the vintage year and is assigned with a normalized score from 0 to 1 based on the first year of operation. The earliest built units, started operation in 1979, receive a score of 0 while the most recently built units, started in 2019, receive a score of 1 (Supplementary Table 1).

Plant size is categorized into four groups, including <300MW, >=300MW (300, 330, and 350MW), >=600MW (600 and 660MW), >=1000MW (1000MW). The categories are based on the commonly adopted combustor sizes. The size groups are given rank scores [1,4] according to the unit sizes ranging from <300MW to >=1000MW. The rank scores [1,4] are normalized into [0,1] to eliminate the issue of data scaling.

Combustion technologies are grouped into ultra-supercritical, supercritical, subcritical, and others, ranked from the most to the least efficient with a decreasing score from 4 to 1. Similarly, the rank scores [1,4] are normalized into [0,1]. Application metric has three categories, industrial self-use, combined heat and power (CHP), and power only. CHP and power only units are given the same rank score to retire while the industrial self-use units are given a lower score (Supplementary Table 2). In the sensitivity analysis of a “CHP-prioritized” method, we assign CHP plants located in northern provinces[1] the highest score at 3, and other CHP plants remain the same score as power only plants at 2.

The application metric score [1,2] is later normalized into [0,1] for algorithm input. Metric description, value range, and assigned scores for technical attributes dimension are shown in Supplementary Table 2.

Supplementary Table 2 Description of metrics for technical attributes and score calculation.

<table>
<tr><th colspan="2">Metrics</th><th>Data Type</th><th colspan="12">Score Assignment</th></tr>
<tr><td rowspan="8">Technical Attributes</td><td rowspan="2">Age</td><td rowspan="2">Quantitative</td><td colspan="12">[1979,2019]</td></tr>
<tr><td colspan="12">[0,1]</td></tr>
<tr><td rowspan="2">Size</td><td rowspan="2">Categorical</td><td colspan="3"><300MW</td><td colspan="3">>=300MW</td><td colspan="3">>=600MW</td><td colspan="3">>=1000MW</td></tr>
<tr><td colspan="3">1</td><td colspan="3">2</td><td colspan="3">3</td><td colspan="3">4</td></tr>
<tr><td rowspan="2">Technology</td><td rowspan="2">Categorical</td><td colspan="3">Other</td><td colspan="3">Subcritical</td><td colspan="3">Supercritical</td><td colspan="3">Ultra-Super</td></tr>
<tr><td colspan="3">1</td><td colspan="3">2</td><td colspan="3">3</td><td colspan="3">4</td></tr>
<tr><td rowspan="2">Application</td><td rowspan="2">Categorical</td><td colspan="4">Self-Use</td><td colspan="4">CHP</td><td colspan="4">Power</td></tr>
<tr><td colspan="4">1</td><td colspan="4">2</td><td colspan="4">2</td></tr>
</table>

2. Profitability

The profitability of a plant is approximated through its current annual gross profit, weighted by capacity. Gross profit is estimated by the difference between the annual revenue and annual cost of the coal-fired power plants in the year of 2015.

$$Net\,profit = Revenue - Cost$$

Equation 1

[1] Hebei, Shanxi, Inner Mongolia, Heilongjiang, Jilin, Liaoning, Shaanxi, Ningxia, Gansu, Qinghai, and Xinjiang.

The annual revenue is estimated by electricity price and amount of electricity generated by the coal-fired power plants.

$$Revenue = P_{elecoal} * Q_{elecoal}$$

Equation 2

Where, $P_{eleccoal}$ represents the electricity (sourced from coal) price by province, RMB (2015)/MWh[5] (NEA, 2016); $Q_{eleccoal}$ is the electricity generated by coal power plants, MWh. $Q_{eleccoal}$ is estimated by the product of coal plant capacity (MW) and operating hours (hr). Plant-level capacity is derived from Global Coal Plant Tracker, and operating hours are from Beijixing Website.[6] For Combined Heat and Power (CHP) plants, heat revenue is estimated of 0.5 of the plant's electricity revenue.

The annual costs of coal-fired power plants are the sum of delivered fuel cost (*coalcost*), variable Operating and Maintenance (O&M) cost (*varOM*), fixed O&M costs (*fixOM*), and additional costs, including environmental costs and tax (*add*), as follows:

$$Cost = coalcost + varOM + fixOM + add$$

Equation 3

Coal is the main fuel to support the operation for coal-fired power plants. It is calculated in Equation 4.

$$coalcost = coalcost_u / \alpha * H$$

Equation 4

coalcost is measured in the price of delivered coal, in which costs of purchasing and transportation are included. Unitary delivered coal price ($coalcost_u$) by province is derived from GCAM (Kim et al., 2006), in RMB/t. α is standard coal consumption rate, referring to lower heating value (LHV), 27,778.62 Btu/t. *H* represents the heat rate, which is dependent on the technology, age, and size of the coal power plants, in Btu/kWh.[7]

$$H = H_{base} * \theta$$

Equation 5

Where, H_{base} denotes the base heat rate, dependent on the technology (Global Coal Plant Tracker). θ is the adjustment multiplier, based on the size and age of the coal power plants, ranging from 1-1.45. The capacity-adjusted multipliers increase when capacity decreases. We

assume the multiplier for plants with capacity ≥ 1000MW as 1, 600MW ≤ capacity < 1000MW as 1.05, 300MW ≤ capacity < 600MW as 1.1, and capacity <300MW as 1.2. Age effects on the heat rate is linear on top of the capacity-adjusted heat rate, which is calculated by $H_{cap} + (age/100 - 0.1)$.[8]

The Operation and Maintenance (O&M) costs include the variable O&M cost (*varOM*) and fixed O&M (*fixOM*) cost. Variable O&M cost refers to long run marginal cost that measures the cost to produce a unit of electric energy, 2.76 $(2015)/MWh in this study; while fixed O&M cost captures the recurring annual cost that occurs regardless of the size or architecture of the power system, 11.03 $(2015)/kW/yr.[9]

$$varOM = varOM_u * Q_{elecoal}$$

Equation 6

$$fixOM = fixOM_u * Capacity$$

Equation 7

Additional costs (add) mainly include environmental costs and tax. Environmental cost is 0.006 RMB/kWh.[10]

Simple [0,1] normalization is applied the quantitative annual gross profit at unit-level to retrieve retirement rank score for profitability dimension. Details of the metric description, value range and corresponding rank score are shown in Supplementary Table 3.

Supplementary Table 3 Score assignment in plant-plant retirement algorithm for profitability metric.

Metrics		Data Type	Score Assignment
Profitability	Annual gross profit	Quantitative	[0.29,259.87]
			[0,1]

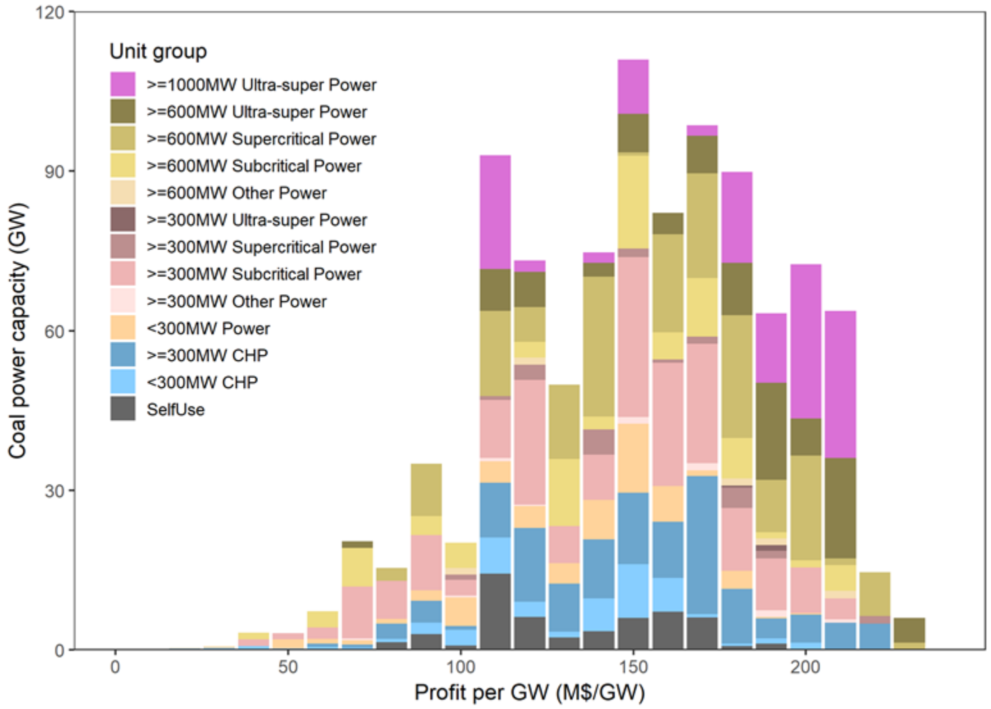

Supplementary Figure 2 Distribution of annual profit per generation

In general, the profitability metric gross profit depends on efficiency—how much coal consumed per electricity generation, operating hour, electricity price, and coal price. Efficiency, or heat rate, is highly associated with the plant's technical attributes, while operating hours, electricity and coal prices vary at the provincial level.

We use a simplified metric to approximate the relative profitability across plants. Other methods, such as the net present value (NPV) or rate of return (RoR), may produce more accurate estimates of the absolute profitability of individual plants. However, these metrics require more input data, such as the initial investment, financial costs, taxes, pollution control costs, and so forth. Since our analysis focuses on the ranking of plants, this simplified metric provides a reasonable approximation suitable for ranking, while acknowledging that it may not represent the absolute profitability of each plant. Because profitability has been the most critical factor in coal plants' shutdown decision-making in the United States, [11] future research could analyze profitability to refine the recommendations for individual Chinese coal plants.

3. Environmental impacts

Our assessment of environmental impacts integrates three elements — 1) global climate change, 2) local air pollution and human health, and 3) water impact.

1) Global climate change

First, to assess the impact of individual coal power unit on global climate change, CO_2 emission rate is estimated at unit-level. Here CO2 emission rate refers to the amount of CO_2 emitted per unit of electricity generation. The annual CO_2 emissions are calculated as follows:

$$Annual\ CO_2 = elecgen * \gamma * H * E * c$$

Equation 8

$$elegen = Capacity * T$$

Equation 9

Where, *Annual CO_2* is the annual CO_2 emissions, in Mt; *elecgen* is the electricity generation, calculated by plant-level capacity (*Capacity*) and operating hours (*T*), in kWh/yr; γ is the conversion coefficient, 1.06×10^{-9} TJ/Btu; *H* represents the heat rate in Btu/kWh; *E* is the carbon content of coal in China, in tC/TJ [12]. Carbon content is dependent on coal type, we used Aden et al. (2008) [13] study to fill the missing data in the Global Coal Plant Tracker database. *c* is constant, which is equal to $12/44\times10^{-9}$.

The CO_2 emission rate depends on plant efficiency as well as the type of coal combusted. However, given the small variation of emission factors across coal types, the CO_2 emission rate is mainly associated with the plant's technical attributes (Supplementary Figure 3). In general, emissions rates increase as a plant gets older, smaller and/or uses a less efficient combustion technology. Coal power units with relatively higher CO_2 emission rates estimated are units which need to be retired first, with lower rank scores assigned.

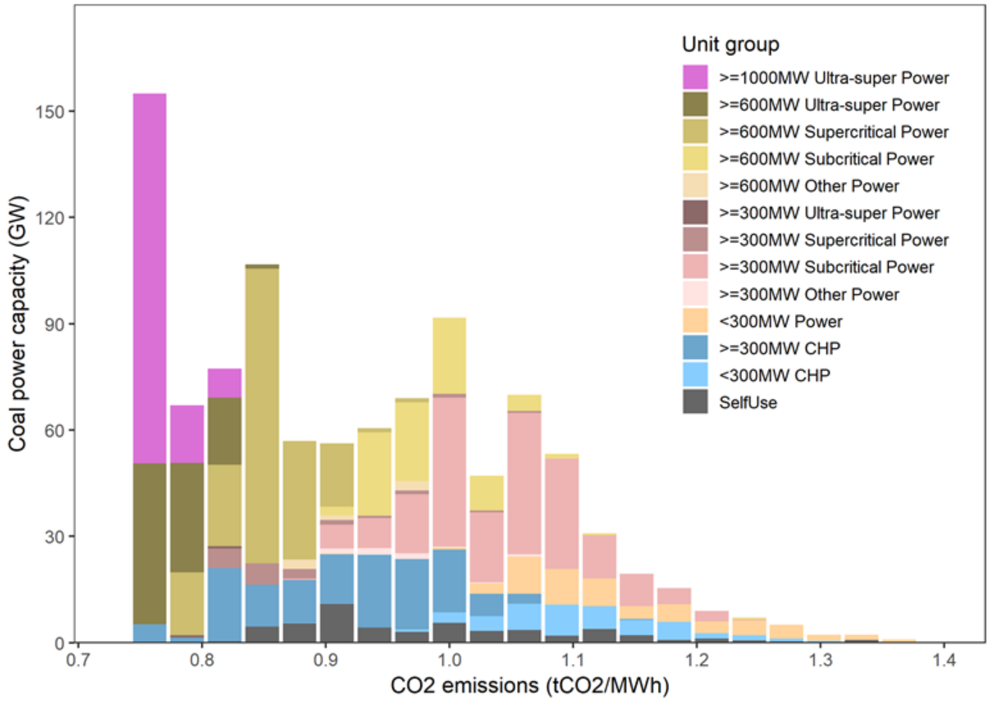

Supplementary Figure 3 Distribution of carbon dioxide emission rate

2) Local air pollution and human health

The local air pollution and human health impact is assessed by looking at the population weighted PM2.5 concentration level at a plant's location. We use the PM2.5 Hindcast Database for China (PHD) (v1.0 of 2016)[14] and UN WPP-Adjusted Population Density from NASA Socioeconomic Data and Applications Center (SEDAC) (v4.11 of 2015).[15] PHD (PM2.5 Hindcast Database) is a database developed and maintained by a team from Tsinghua University, Beijing, China, that provides historical PM2.5 estimates across China, during 2000-2016, in a regular grid of 0.1° × 0.1°. PHD used a machine learning approach to assemble datasets from multiple sources, including MODIS satellite measurements of aerosol, CMAQ modeling outputs based on MEIC historical emission inventories and many other spatiotemporal variables, and hindcasted the daily PM2.5 concentrations from 2000 to 2016 in China. Unit-level PM2.5 exposure is retrieved from the PM2.5 exposure map with national coverage using coordinates of individual units. Units located in highly polluted and highly-populated areas, which can be indicated by higher PM2.5 exposure level, are given lower rank scores.

The issue of air pollution poses a serious threat to Chinese public health and is mainly contributed from burning coal. In the past few years, the majority of coal-fired power plants in China implemented local air pollution control technologies and significantly lowered their SO_2, NO_x, and

primary PM emissions.[16] Although this has reduced some urgency in certain locations for reducing local air pollution immediately, coal plants will remain a critical piece in the longer term effort to further improve air quality in line with the WHO guidelines on the safer $PM_{2.5}$ concentration level. Therefore, our metric, the annual $PM_{2.5}$ concentration [17] weighted by population density, [18] represents the potential health benefit by closing coal units in that gridded cell. The original data on PM2.5 is ranged from 1.5 to 171.6 μg/m^3, while the logarithmic population weighted annual PM2.5 is ranged between 0 to 43.90 μg/m^3. Both are comparable to the literature[19,20]. The higher the current health impact, the larger the marginal benefit of shutting down a coal plant, assuming the same level of air pollution control implemented. For example, plants located along the densely populated Beijing-to-Shanghai corridor and several major cities such as Guangzhou and Chongqing, will receive a lower score on this metric (Supplementary Figure 4a).

3) Water risk

Water impact is estimated with the water risk level of a plant's location using a similar method. The water risk score applied in our research is from Aqueduct Global Maps (v2.1 of 2010) [21], derived from a framework of 12 global water-related risk indicators. The Aqueduct Global Maps 2.1 took indicators of water quantity, water variability, water quality, public awareness of water issues, access to water, and ecosystem vulnerability into account and grouped them into one overall score. This Water Risk Index (WRI) provides a good representation of the physical, regulatory and reputational water risk level. The well-defined comprehensive water risk index (WRI) of a given unit's location is used to indicate the potential reduction in local water impacts by closing that coal unit. It represents the potential reduction in water impact by closing coal units in that gridded cell. Therefore, units in regions facing more severe water scarcity receive lower rank scores. In particular, the northern provinces face more severe water crisis due to limited water resources and water pollution problem, therefore plants located in those regions will receive a lower score on this metric (Supplementary Figure 4b).

a

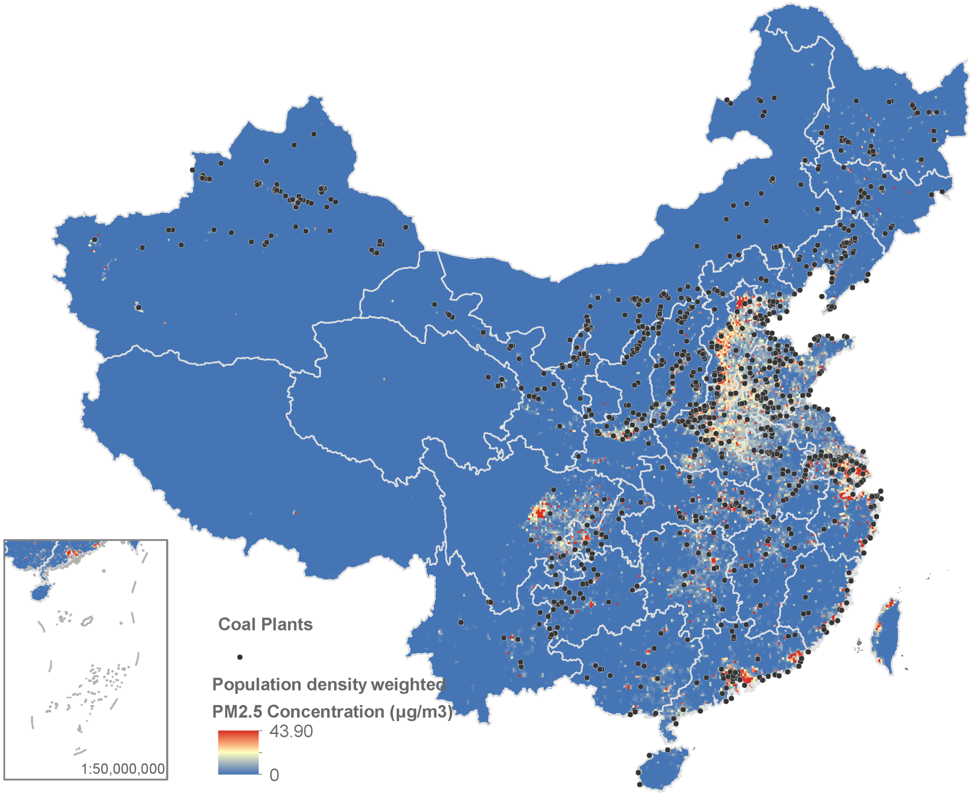

1:50,000,000
Coal Plants
Population density weighted
PM2.5 Concentration (μg/m3)
43.90
0

b

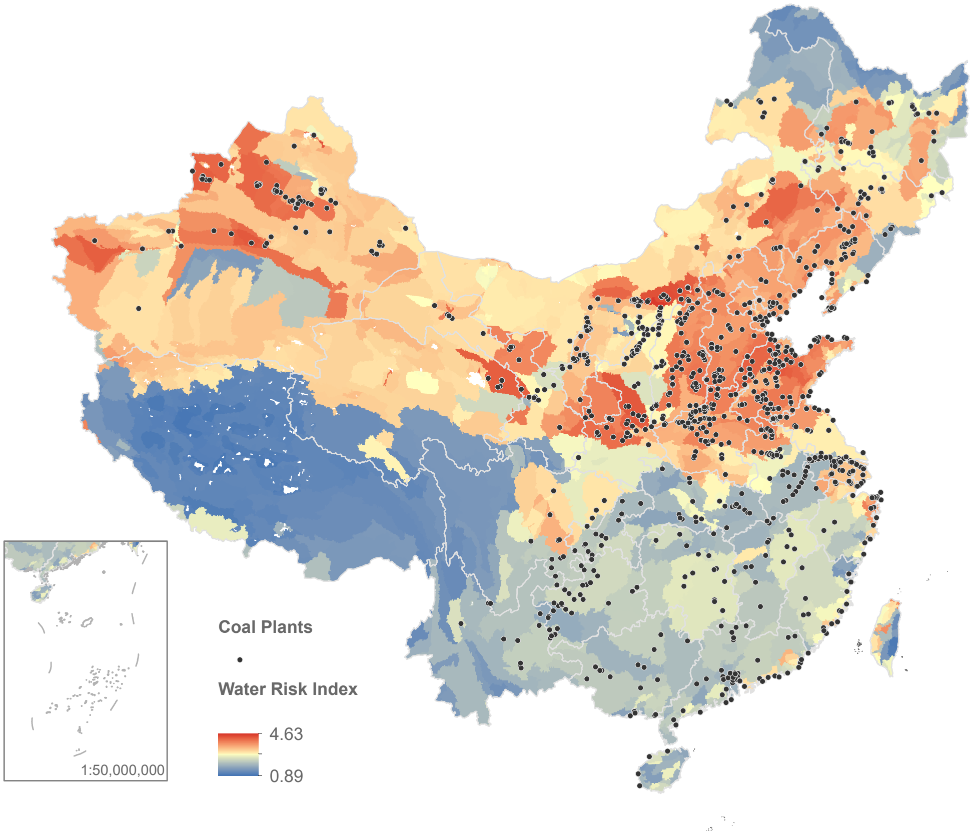

1:50,000,000
Coal Plants
Water Risk Index
4.63
0.89

Supplementary Figure 4 Environmental impacts of existing coal plants. (a) Local air pollution and health impact at operating coal plants' locations: Higher population weighted PM2.5 concentration level is associated with a lower score for plants in that gridded cell, indicating higher marginal health benefit by retiring those units. The original data on PM2.5 is ranged from 1.5 to 171.6 μg/m^3, while the logarithmic population weighted annual PM2.5 is ranged between 0 to 43.90 μg/m^3. (b) Local water impact at operating coal plants' location: Higher water risk level is associated with a lower score for units in that gridded cell, indicating higher marginal water benefit by closing those plants. Blank spots are missing data from the Aqueduct dataset – e.g. water bodies that do not have a water risk index. These places do not have operating coal plants.

Detailed metric description and score assignment for the three environmental impact metrics are listed in Supplementary Table 4.

Supplementary Table 4 Score assignment in plant-plant retirement algorithm for metrics describing the dimension of environmental impacts.

Metrics		Data Type	Score Assignment
Environmental Impacts	CO_2 emission rate	Quantitative	[0.76,1.39]
			[1,0]
	Local air pollution	Quantitative	[0,23.44]
			[1,0]
	Water impact	Quantitative	[1.40,4.63]
			[1,0]

Supplementary Figures

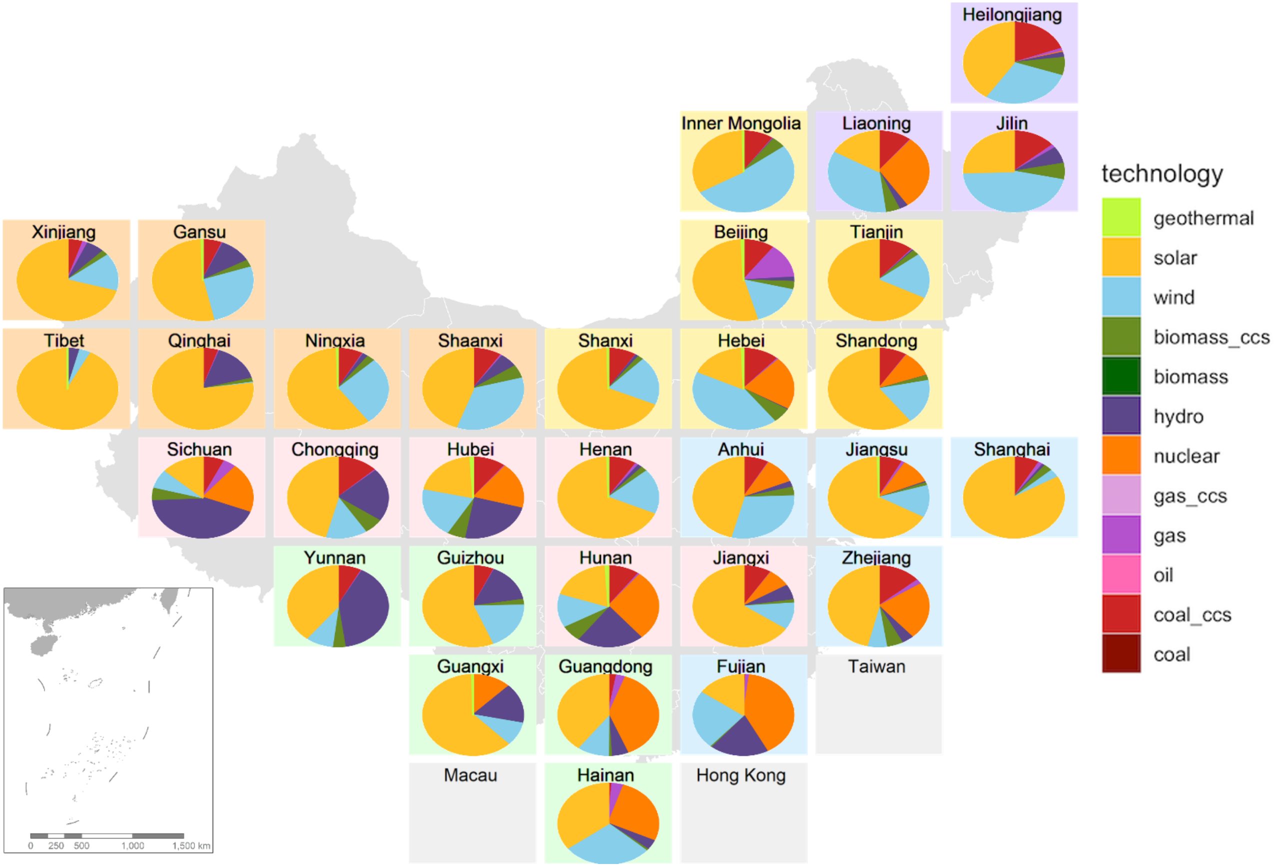

Supplementary Figure 5 Electricity generation share by technology in 2050 by province under 1.5°C.

a

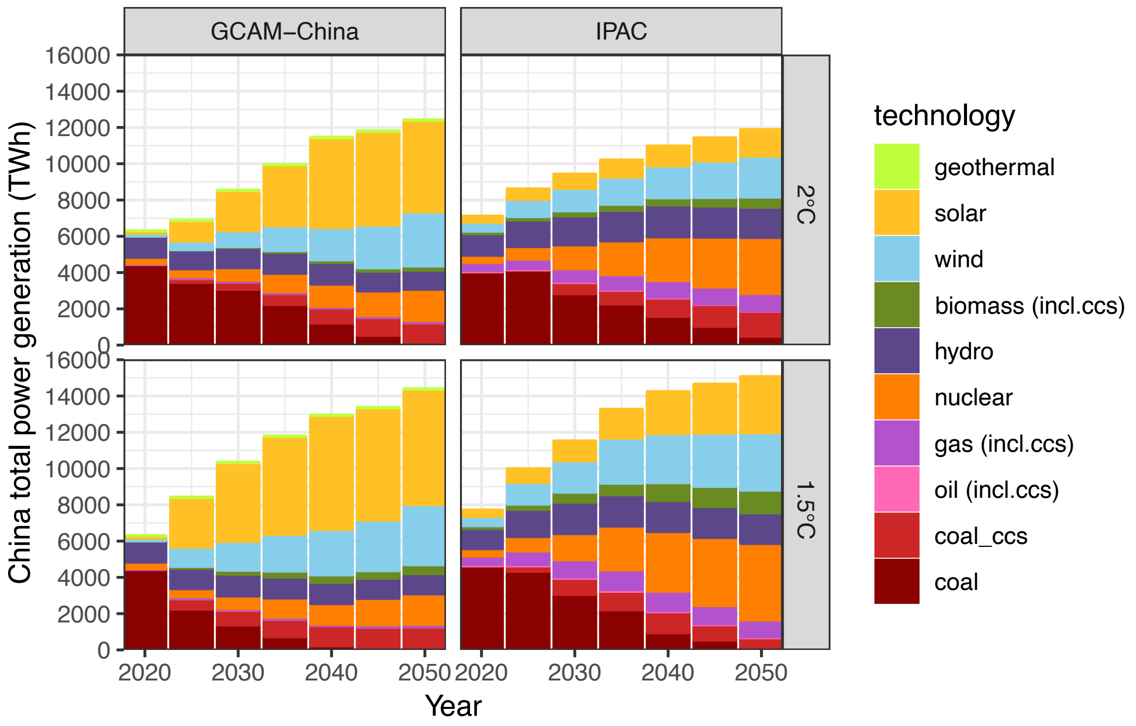
GCAM–China
IPAC
2°C
1.5°C
China total power generation (TWh)
Year
technology
geothermal
solar
wind
biomass (incl.ccs)
hydro
nuclear
gas (incl.ccs)
oil (incl.ccs)
coal_ccs
coal

b

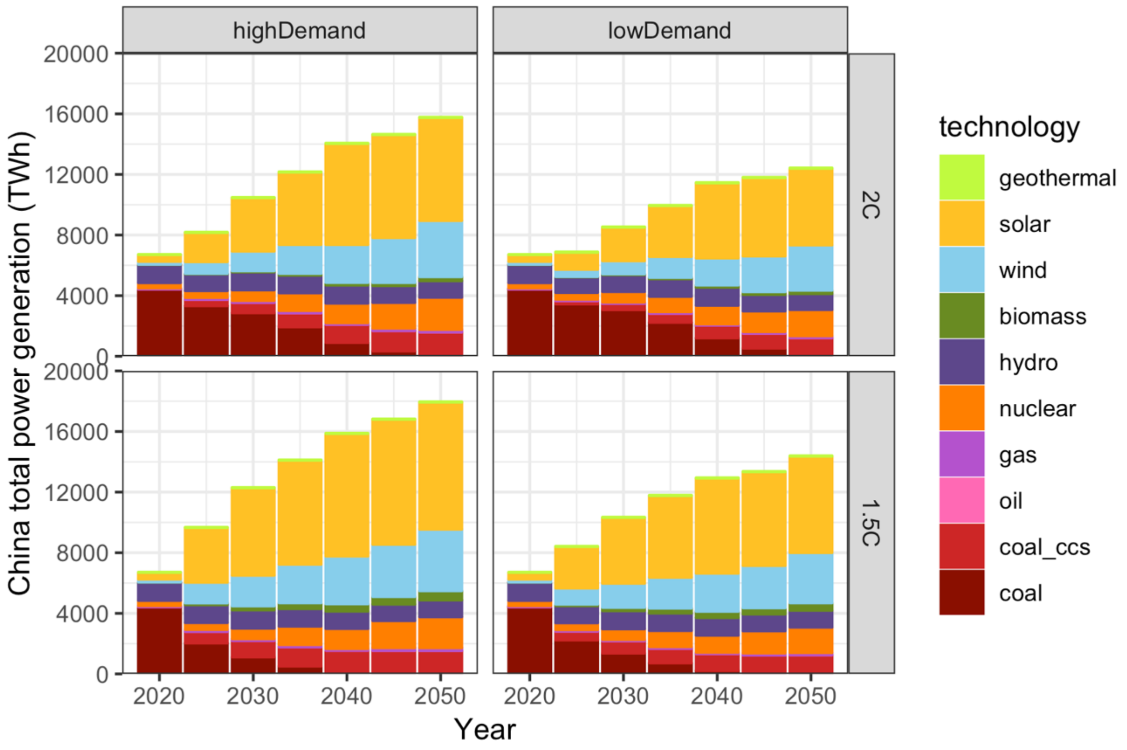
highDemand
lowDemand
2C
1.5C
China total power generation (TWh)
Year
technology
geothermal
solar
wind
biomass
hydro
nuclear
gas
oil
coal_ccs
coal

c

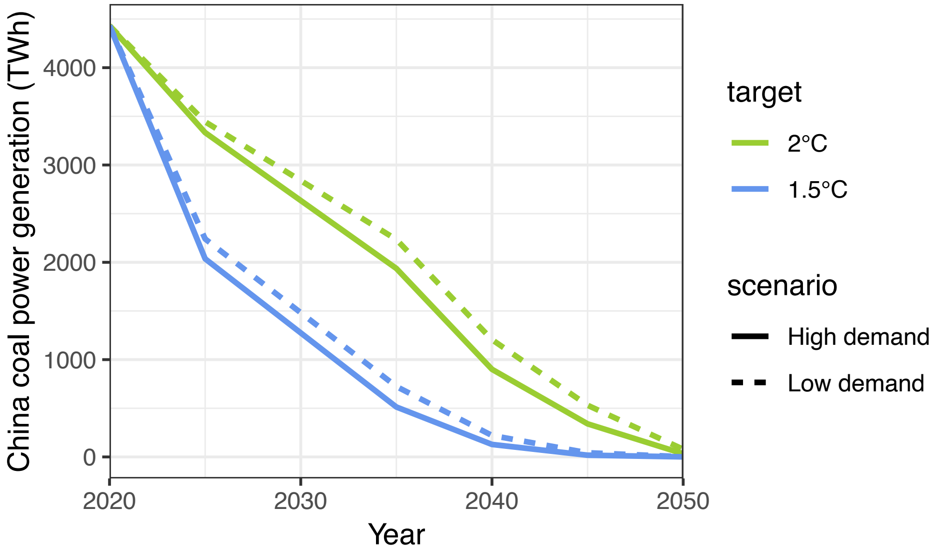

d

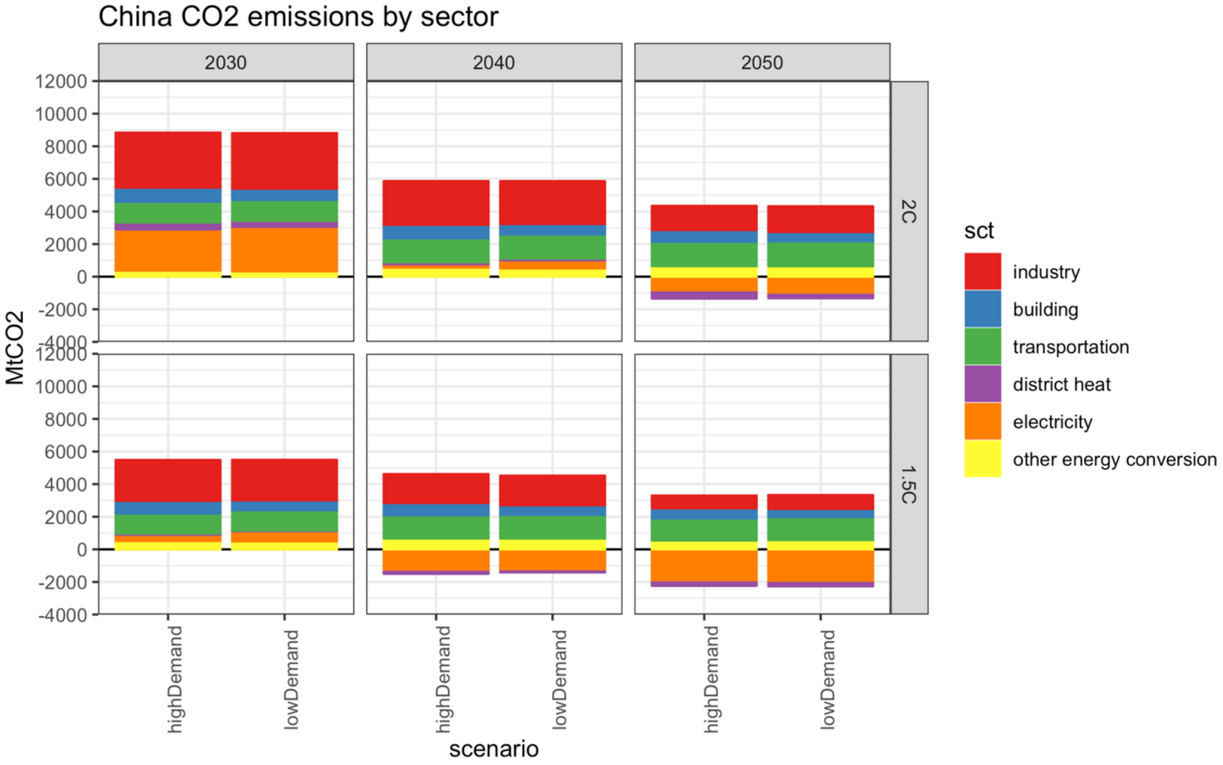

Supplementary Figure 6 National power generation under 1.5°C and 2°C: (a) by technology and by model, (b) by technology and by different demand scenario, (c) by conventional coal-fired power plants under different demand scenarios. (d) CO_2 emissions by different sectors under different demand scenarios

a

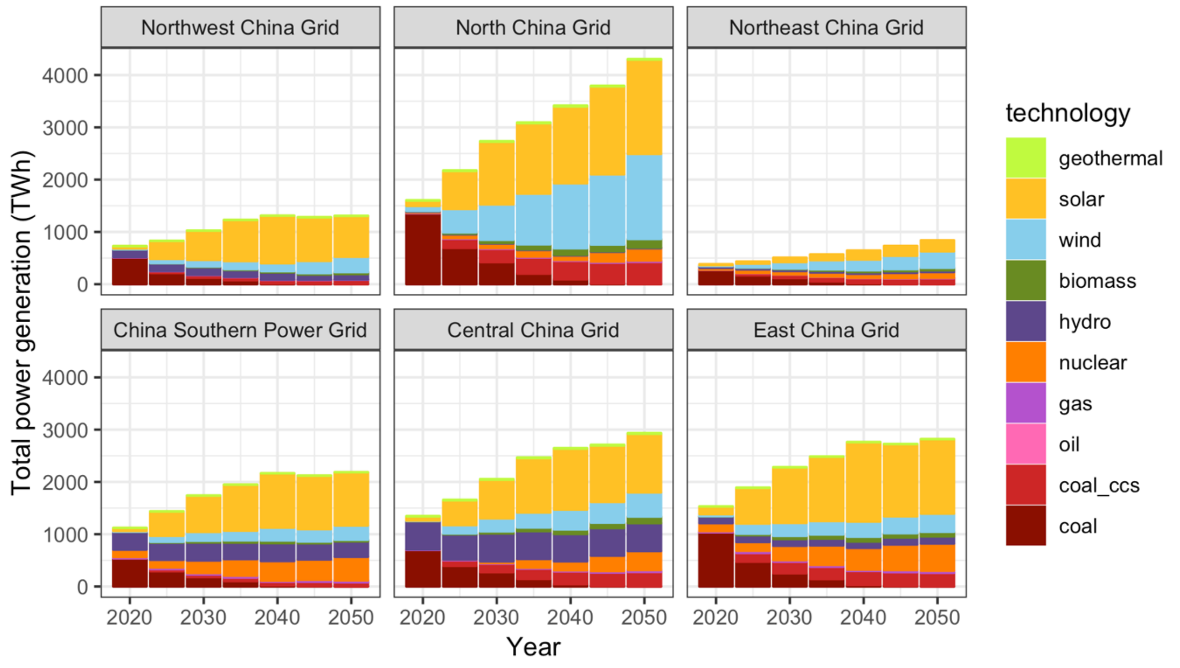

b

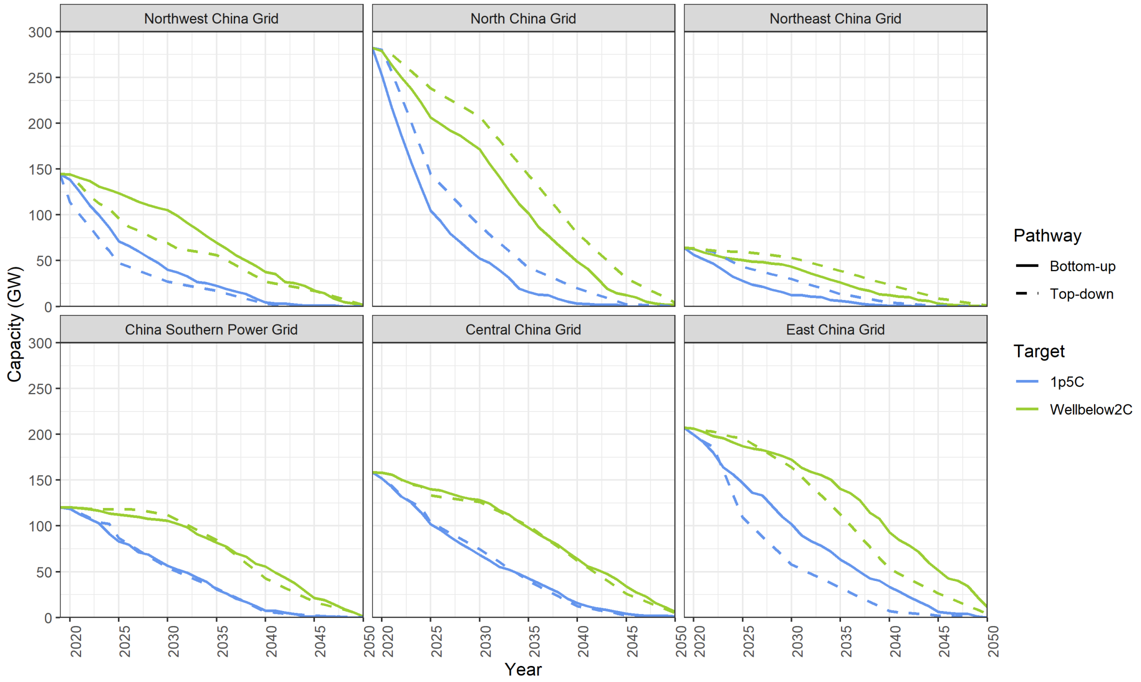

Supplementary Figure 7 Electricity by grid region: (a) generation by technology under 1.5°C; (b) conventional coal-fired power capacity with the bottom up plant-by-plant pathway (solid line) and the top-down GCAM-China modeling results (dashed line) under 1.5°C (blue) and 2°C (green).

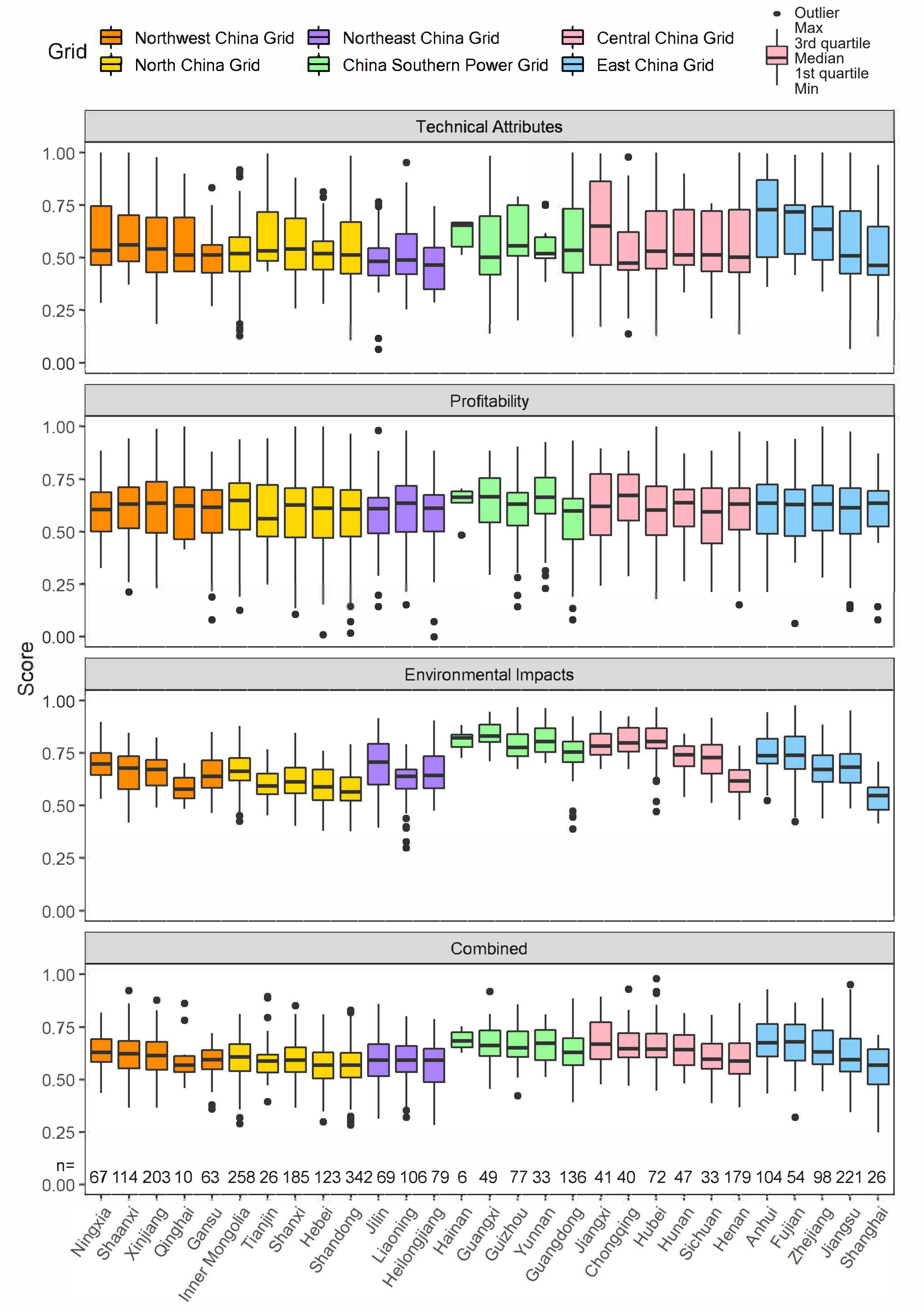

Supplementary Figure 8 Provincial distribution of the dimensional and combined scores.

a

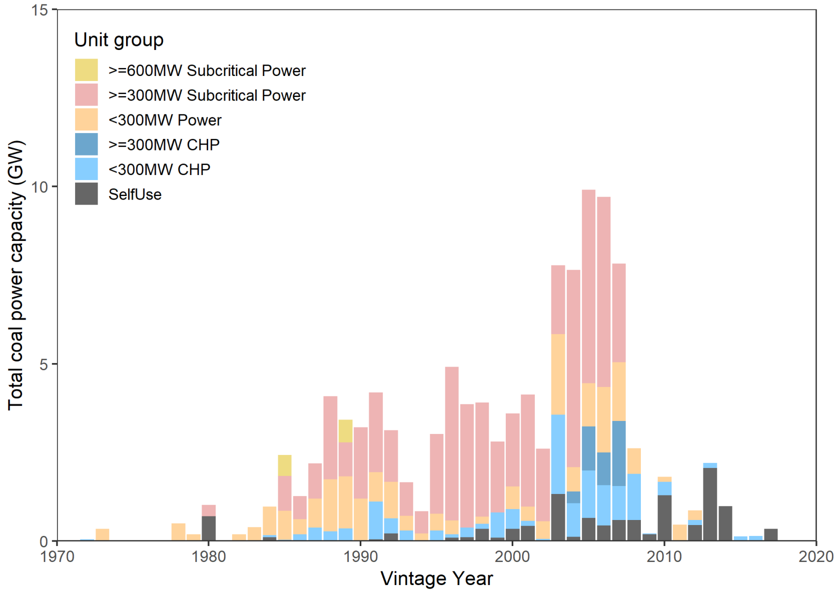

b

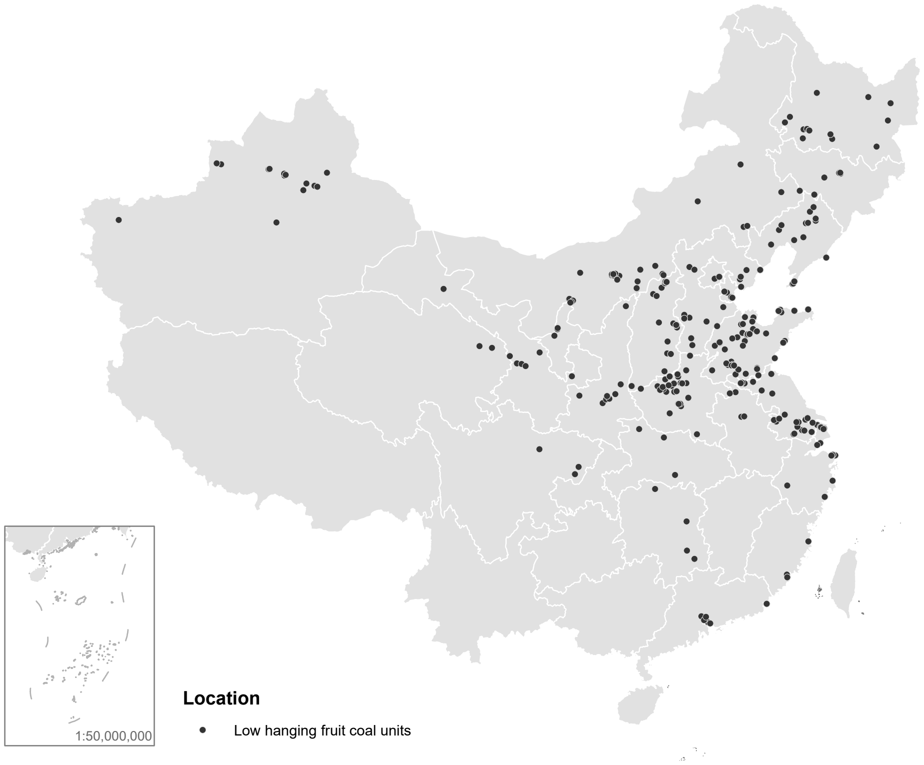

Supplementary Figure 9 National distribution of existing coal capacity of low-hanging fruit coal units by technical attributes (a) and their spatial locations (b).

Supplementary Table 5 Number of units and total capacity of low-hanging fruit plants in each province

Province	Number of units		Installed capacity	
	Unit	Percentage of provincial total	MW	Percentage of provincial total
Shandong	98	29%	18,586	20%
Inner Mongolia	50	19%	11,710	14%
Henan	48	27%	10,005	15%
Jiangsu	46	21%	9,489	13%
Hebei	36	29%	9,910	26%
Shanxi	36	19%	8,425	14%
Xinjiang	30	15%	4,785	9%
Heilongjiang	29	37%	4,295	23%
Liaoning	27	25%	5,110	18%
Jilin	18	26%	3,076	18%
Zhejiang	16	16%	2,602	6%
Gansu	14	22%	3,485	17%
Shaanxi	14	12%	3,515	9%
Shanghai	9	35%	2,715	23%
Guangdong	9	7%	1,770	3%
Fujian	8	15%	2,000	7%
Tianjin	8	31%	1,956	17%
Hunan	7	15%	1,950	10%
Ningxia	6	9%	1,980	7%
Sichuan	6	18%	1,860	16%
Anhui	4	4%	1,480	3%
Hubei	4	6%	220	1%
Qinghai	3	30%	405	13%
Guizhou	0	0%	0	0%
Jiangxi	0	0%	0	0%
Guangxi	0	0%	0	0%
Chongqing	0	0%	0	0%
Yunnan	0	0%	0	0%
Hainan	0	0%	0	0%
National total	*526*	*18%*	*111,329*	*11%*

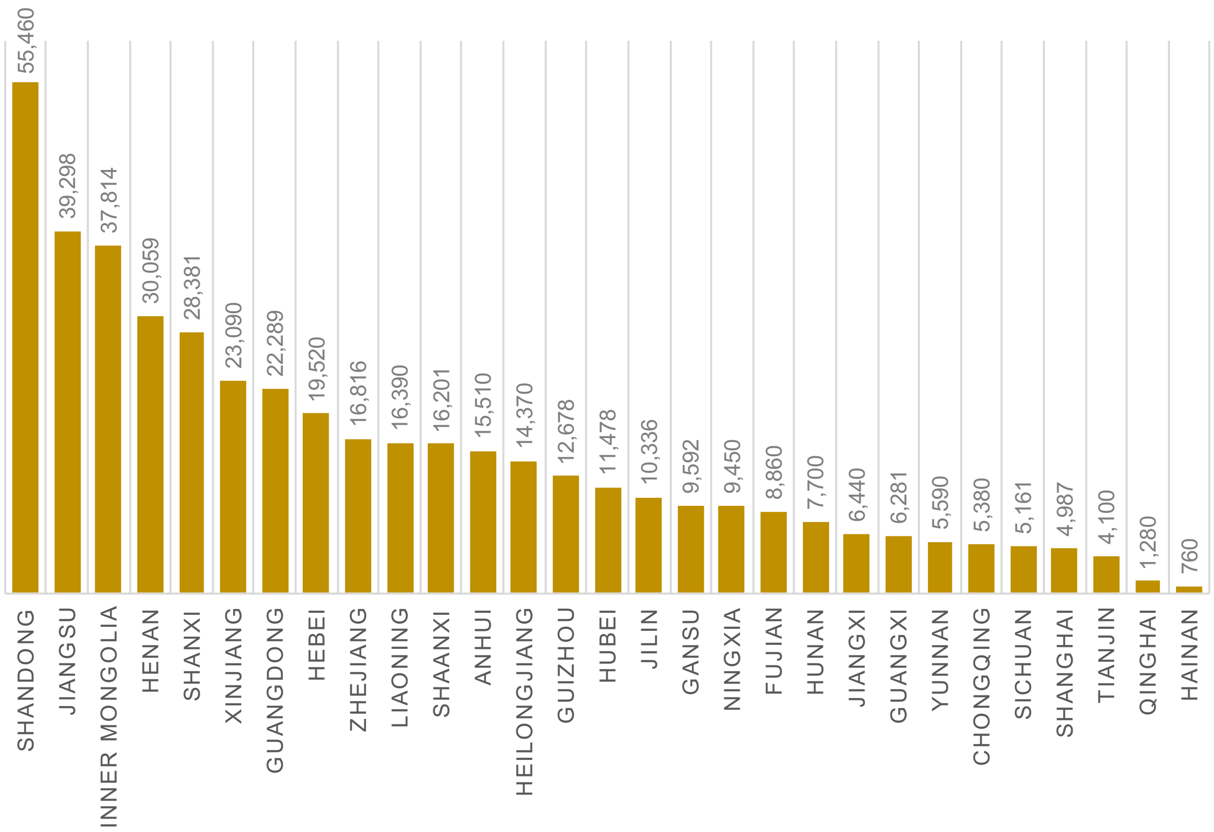

Supplementary Figure 10 Total employment of coal power plants, by province. Older plants implemented before 2007 tend to have a much larger number of workers per unit of capacity.

a

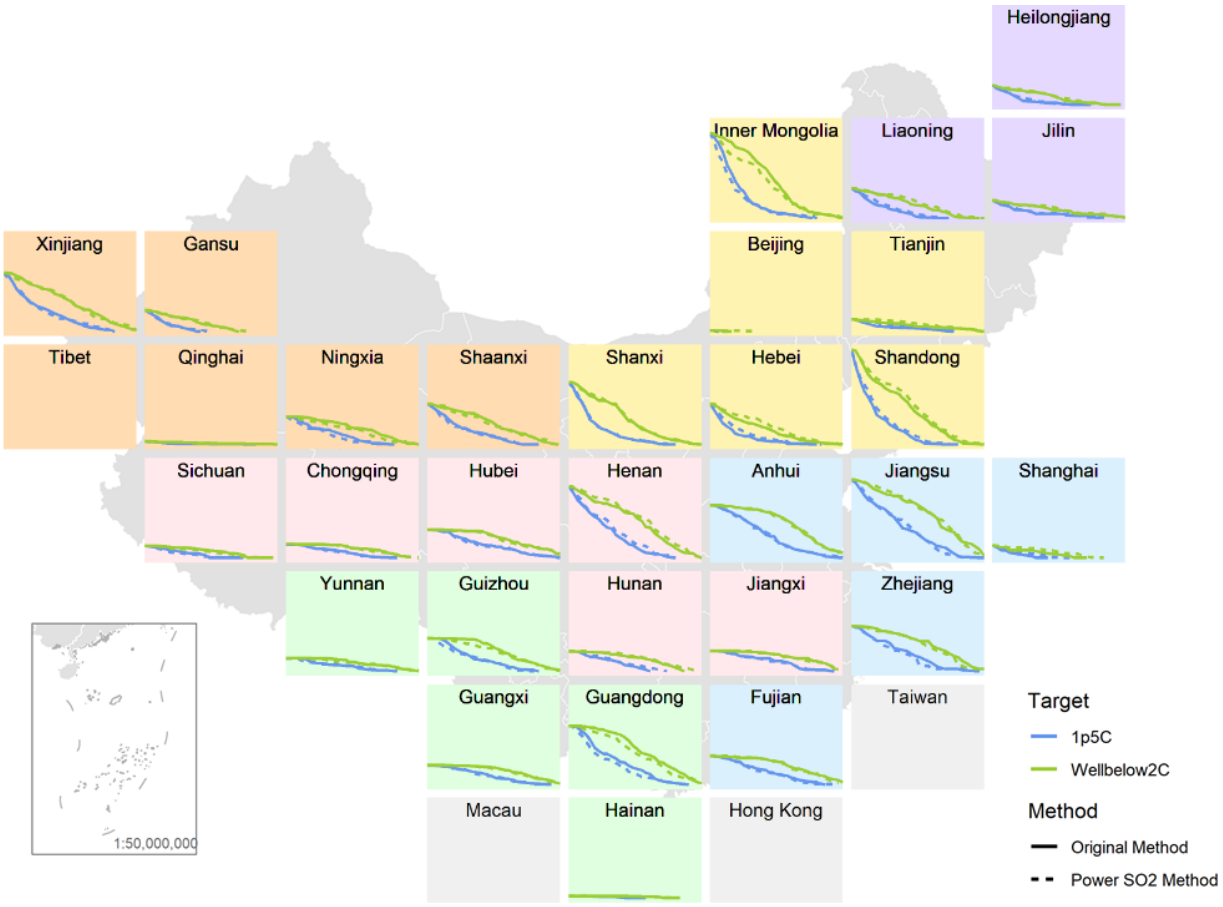

Heilongjiang
Inner Mongolia
Liaoning
Jilin
Xinjiang
Gansu
Beijing
Tianjin
Tibet
Qinghai
Ningxia
Shaanxi
Shanxi
Hebei
Shandong
Sichuan
Chongqing
Hubei
Henan
Anhui
Jiangsu
Shanghai
Yunnan
Guizhou
Hunan
Jiangxi
Zhejiang
Guangxi
Guangdong
Fujian
Taiwan
Macau
Hainan
Hong Kong
1:50,000,000
Target
1p5C
Wellbelow2C
Method
Original Method
Power SO2 Method

b

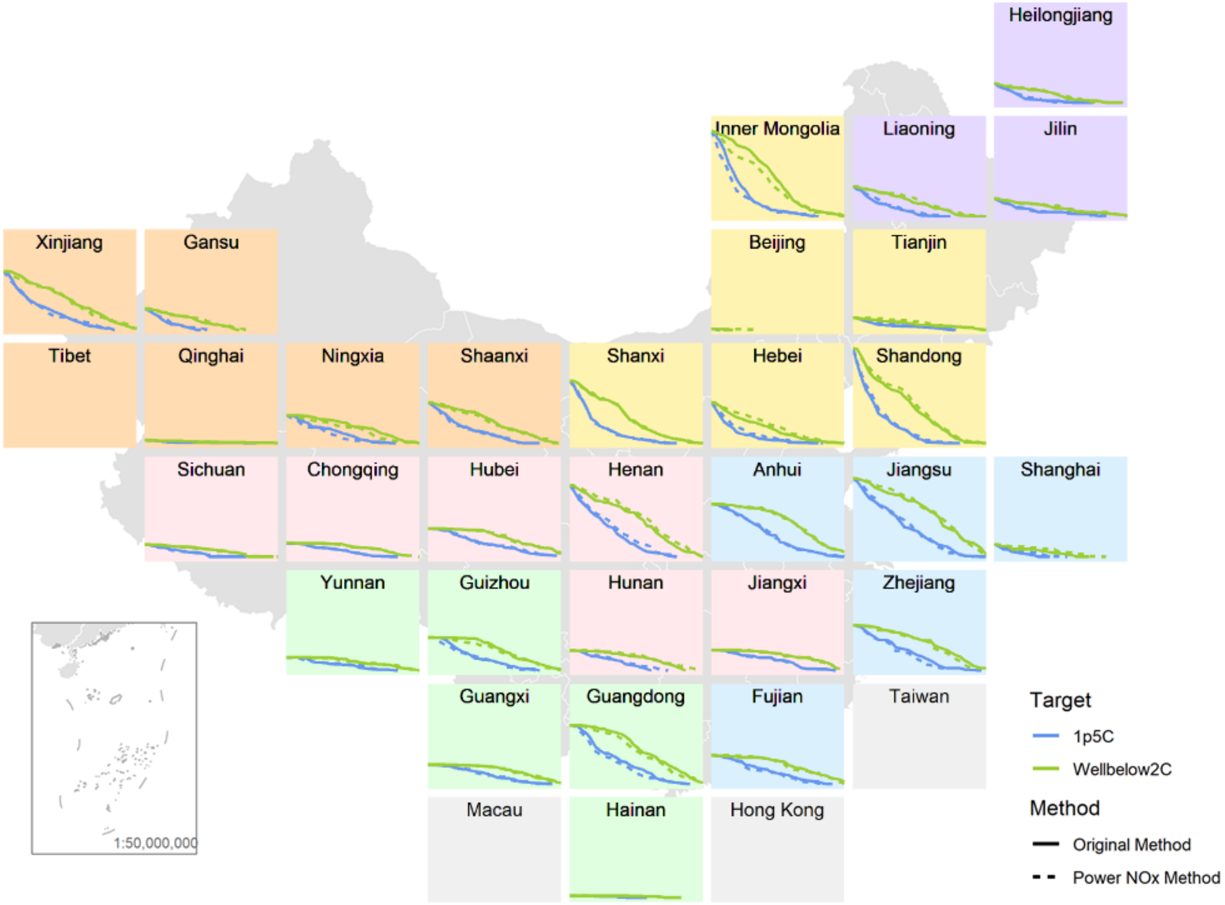
Heilongjiang
Inner Mongolia
Liaoning
Jilin
Xinjiang
Gansu
Beijing
Tianjin
Tibet
Qinghai
Ningxia
Shaanxi
Shanxi
Hebei
Shandong
Sichuan
Chongqing
Hubei
Henan
Anhui
Jiangsu
Shanghai
Yunnan
Guizhou
Hunan
Jiangxi
Zhejiang
Guangxi
Guangdong
Fujian
Taiwan
Macau
Hainan
Hong Kong
1:50,000,000
Target
1p5C
Wellbelow2C
Method
Original Method
Power NOx Method

c

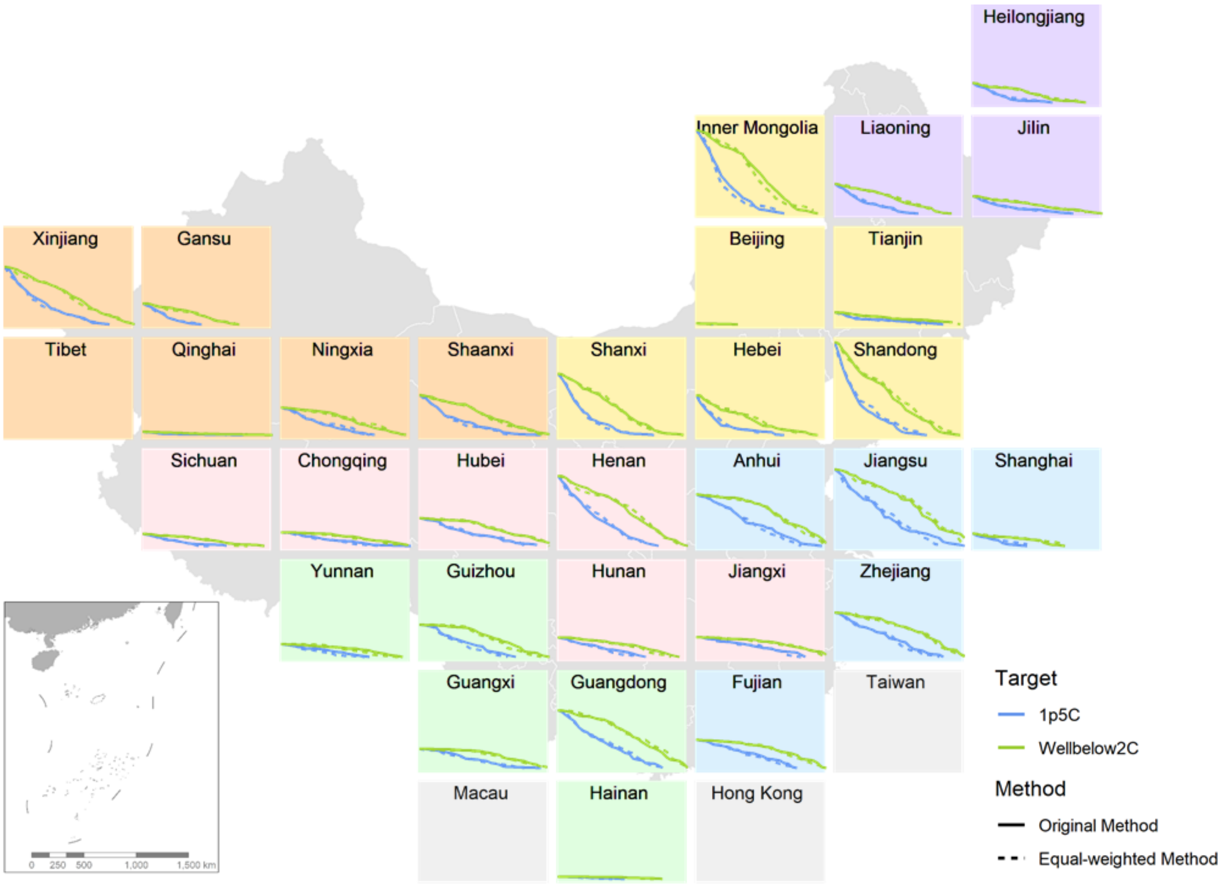

Heilongjiang
Inner Mongolia
Liaoning
Jilin
Xinjiang
Gansu
Beijing
Tianjin
Tibet
Qinghai
Ningxia
Shaanxi
Shanxi
Hebei
Shandong
Sichuan
Chongqing
Hubei
Henan
Anhui
Jiangsu
Shanghai
Yunnan
Guizhou
Hunan
Jiangxi
Zhejiang
Guangxi
Guangdong
Fujian
Taiwan
Macau
Hainan
Hong Kong
Target
1p5C
Wellbelow2C
Method
Original Method
Equal-weighted Method

d

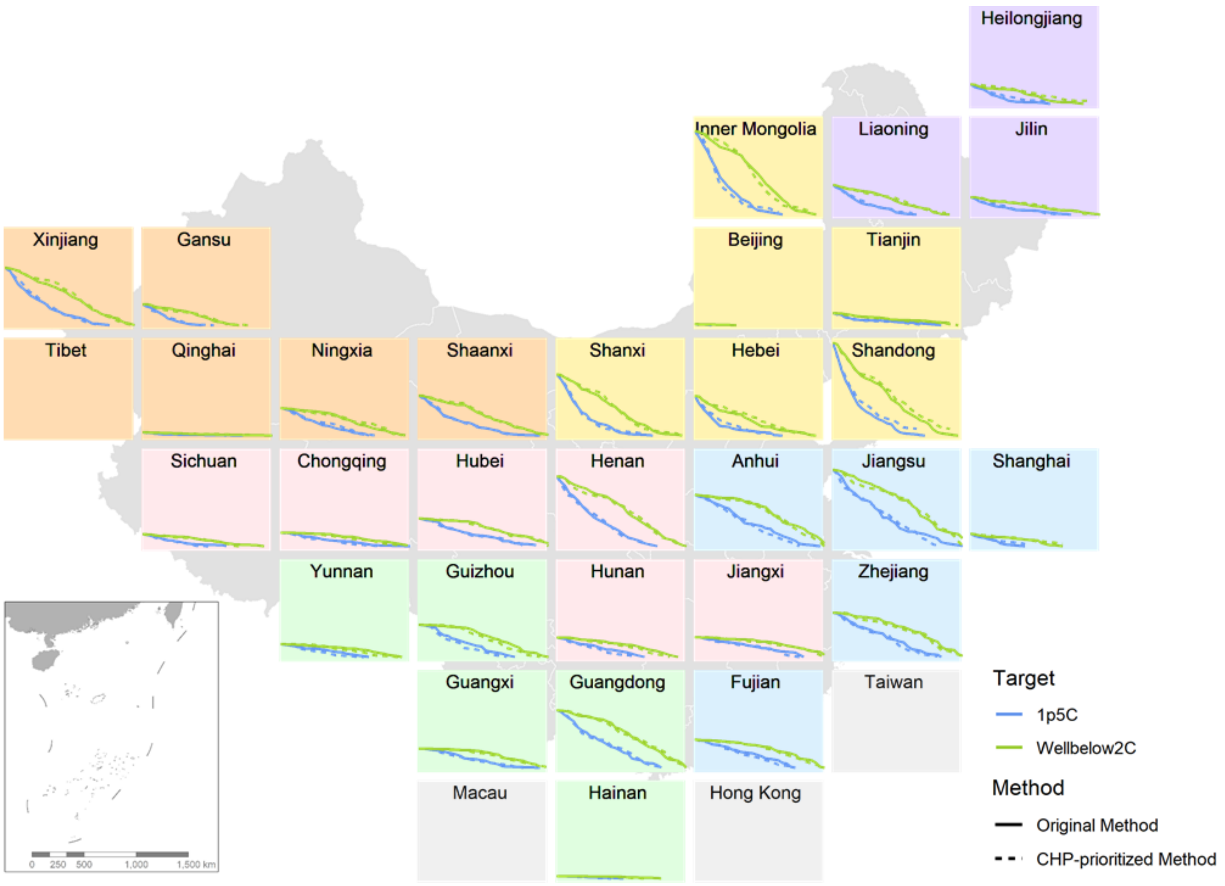

Supplementary Figure 11 Comparison of provincial retirement pathways retrieved using different weighting methods: (a) power sector SO2 contribution method, (b) power sector NOx contribution method, (c) equal-weighted method, and (d) CHP-prioritized method.

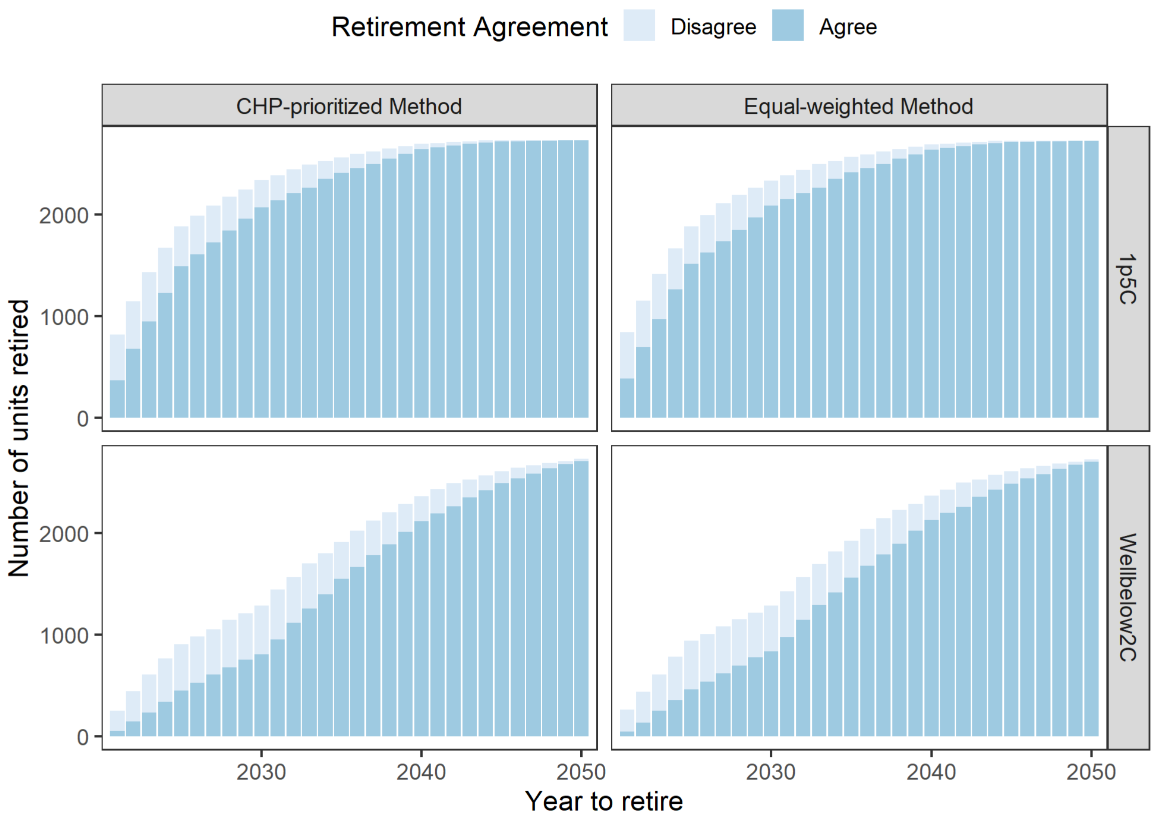

Supplementary Figure 12 Retirement agreement between weighting methods at unit level.

Supplementary Reference

[1] Zhang, Q., Streets, D. G., Carmichael, G. R., He, K. B., Huo, H., Kannari, A., Klimont, Z., Park, I. S., Reddy, S., Fu, J. S., Chen, D., Duan, L., Lei, Y., Wang, L. T., and Yao, Z. L. (2009), Asian emissions in 2006 for the NASA INTEX-B mission, Atmos. Chem. Phys., 9, 5131-5153.

[2] Zheng, B., Huo, H., Zhang, Q., Yao, Z. L., Wang, X. T., Yang, X. F., Liu, H., and He, K. B.: High-resolution mapping of vehicle emissions in China in 2008, Atmos. Chem. Phys., 14, 9787-9805, doi:10.5194/acp-14-9787-2014, 2014.

[3] Li, M., Zhang, Q., Streets, D. G., He, K. B., Cheng, Y. F., Emmons, L. K., Huo, H., Kang, S. C., Lu, Z., Shao, M., Su, H., Yu, X., and Zhang, Y.: Mapping Asian anthropogenic emissions of non-methane volatile organic compounds to multiple chemical mechanisms, Atmos. Chem. Phys., 14, 5617-5638, doi:10.5194/acp-14-5617-2014, 2014.

[4] Liu, F., Zhang, Q., Tong, D., Zheng, B., Li, M., Huo, H., and He, K. B.: High-resolution inventory of technologies, activities, and emissions of coal-fired power plants in China from 1990 to 2010, Atmos. Chem. Phys., 15, 13299-13317, doi:10.5194/acp-15-13299-2015, 2015.

[5] National Energy Administration of the People's Republic of China (NEA). (2016). National Energy

Administration Released 2015 Electricity Consumption Data.

[6] http://news.bjx.com.cn/html/20190214/962680.shtml

[7] Tong, D., Zhang, Q., Davis, S. J., Liu, F., Zheng, B., Geng, G., ... & Streets, D. G. (2018). Targeted emission reductions from global super-polluting power plant units. Nature Sustainability, 1(1), 59.

[8] International Energy Agency (IEA). (2012). Technology Roadmaps: High-efficiency, low-emissions coal-fired power generation. p.17.

[9] Miao, B. (2015). Outlook of Power Generation Technology Cost in China. Norwgian School of Economics, Bergen.

[10] Yuan, J., Hu, Z., Zhang, W. (2016). National Development and Reform Commission (NDRC, 2014). The 2005 National GHG Inventory of China.

[11] Fleischman, L., Cleetus, R., Deyette, J., Clemmer,S,& Frenkel, S. Ripe for retirement: An economic analysis of the US coal fleet. *The Electricity Journal* 26(10): 51-63 (2013).

[12] National Development and Reform Commission (NDRC). (2014). The 2005 National GHG Inventory of China.

[13] Aden, N. T., Fridley, D. G., & Zheng, N. (2008). Outlook and Challenges for Chinese Coal (No. LBNL-4772E). Lawrence Berkeley National Lab.(LBNL), Berkeley, CA (United States).

[14] Xue T, Zheng Y, Tong D, Zheng B, Li X, Zhu T, Zhang Q. (2018). Spatiotemporal continuous estimates of PM2.5.

[15] NASA Socioeconomic Data and Applications Center (SEDAC): UN WPP-Adjusted Population Density, v4.11 (2015).

[16] Tang, L., Qu, J., Mi, Z., Bo, X., Chang, X., Anadon, L. D., ... & Zhao, X. Substantial emission reductions from Chinese power plants after the introduction of ultra-low emissions standards. Nature Energy, 4(11), 929-938 (2019).

[17] Xue, T., Zheng, Y., Tong, D., Zheng, B., Li, X., Zhu, T., & Zhang, Q. Spatiotemporal continuous estimates of PM2. 5 concentrations in China, 2000–2016: A machine learning method with inputs from satellites, chemical transport model, and ground observations. *Environment International*, 123: 345-357 (2019).

[18] Center for International Earth Science Information Network (CIESIN), Columbia University. Gridded Population of the World, Version 4 (GPWv4): Population Density Adjusted to Match 2015 Revision of UN WPP Country Totals, Revision 11. Palisades, NY: NASA Socioeconomic Data and Applications Center (SEDAC). (2018) https://doi.org/10.7927/H4F47M65.

[19] Zhang, Q. *et al.* Drivers of improved PM2.5 air quality in China from 2013 to 2017. *Proc. Natl. Acad. Sci. U. S. A.* (2019). doi:10.1073/pnas.1907956116

[20] Zeng, M., Du, J. & Zhang, W. Spatial-Temporal Effects of PM(2.5) on Health Burden: Evidence from China. *Int. J. Environ. Res. Public Health* **16**, 4695 (2019).

[21] Gassert, F., M. Luck, M. Landis, P. Reig, and T. Shiao. (2014). Aqueduct Global Maps 2.1: Constructing Decision-Relevant Global Water Risk Indicators. Working Paper. Washington, DC: World Resources Institute. Available online at http://www.wri.org/publication/aqueduct-globalmaps-21-indicators
