# Supplementary material for: A plant-by-plant strategy for high-ambition coal power phaseout in China
Source: Nat Commun. 2021 Mar 16;12:1468. doi: 10.1038/s41467-021-21786-0 (PMC7966364; doi:10.1038/s41467-021-21786-0)
Supplement: Supplementary file 2 — Reporting Summary [file 41467_2021_21786_MOESM2_ESM.pdf]

nature research

Corresponding author(s): Ryna Yiyun Cui

Last updated by author(s): Dec 19, 2020

Reporting Summary

Nature Research wishes to improve the reproducibility of the work that we publish. This form provides structure for consistency and transparency in reporting. For further information on Nature Research policies, see our Editorial Policies and the Editorial Policy Checklist.

Statistics

For all statistical analyses, confirm that the following items are present in the figure legend, table legend, main text, or Methods section.

n/a	Confirmed	
x	☐	The exact sample size (n) for each experimental group/condition, given as a discrete number and unit of measurement
x	☐	A statement on whether measurements were taken from distinct samples or whether the same sample was measured repeatedly
x	☐	The statistical test(s) used AND whether they are one- or two-sided<br>*Only common tests should be described solely by name; describe more complex techniques in the Methods section.*
x	☐	A description of all covariates tested
x	☐	A description of any assumptions or corrections, such as tests of normality and adjustment for multiple comparisons
x	☐	A full description of the statistical parameters including central tendency (e.g. means) or other basic estimates (e.g. regression coefficient) AND variation (e.g. standard deviation) or associated estimates of uncertainty (e.g. confidence intervals)
x	☐	For null hypothesis testing, the test statistic (e.g. F, t, r) with confidence intervals, effect sizes, degrees of freedom and P value noted<br>*Give P values as exact values whenever suitable.*
x	☐	For Bayesian analysis, information on the choice of priors and Markov chain Monte Carlo settings
x	☐	For hierarchical and complex designs, identification of the appropriate level for tests and full reporting of outcomes
x	☐	Estimates of effect sizes (e.g. Cohen's d, Pearson's r), indicating how they were calculated

Our web collection on statistics for biologists contains articles on many of the points above.

Software and code

Policy information about availability of computer code

Data collection	We did not use any code or software for data collection. We did manual Google search for key words (i.e. individual project name).
Data analysis	We used R (3.6.3) program to process data and generate charts. We used the Global Change Analysis Model (GCAM-China) to simulate the scenarios. GCAM is an open source integrated assessment model, available at: https://github.com/JGCRI/gcam-core/releases.

For manuscripts utilizing custom algorithms or software that are central to the research but not yet described in published literature, software must be made available to editors and reviewers. We strongly encourage code deposition in a community repository (e.g. GitHub). See the Nature Research guidelines for submitting code & software for further information.

Data

Policy information about availability of data

All manuscripts must include a data availability statement. This statement should provide the following information, where applicable:

- Accession codes, unique identifiers, or web links for publicly available datasets
- A list of figures that have associated raw data
- A description of any restrictions on data availability

Unit-level data of Chinese coal-fired power plants used in this analysis are from the Global Coal Plant Tracker dataset (Global Energy Monitor, Global Coal Plant Tracker, Jan 2019. Available at https://endcoal.org/global-coal-plant-tracker/). Other data used are cited in Supplementary Table 1.

Field-specific reporting

Please select the one below that is the best fit for your research. If you are not sure, read the appropriate sections before making your selection.

☐ Life sciences ☐ Behavioural & social sciences ☒ Ecological, evolutionary & environmental sciences

For a reference copy of the document with all sections, see nature.com/documents/nr-reporting-summary-flat.pdf

Ecological, evolutionary & environmental sciences study design

All studies must disclose on these points even when the disclosure is negative.

Study description	We evaluate the performance of individual existing coal-fired power plants in China, by calculating a combined score of multiple technical, economic, and environmental criteria.
Research sample	We used the entire population data of existing coal-fired power plants in China. The dataset covers 1,037 operating coal plants, nearly 3,000 individual generators, a total of 980 GW. According to China Electricity Council (CEC), the total capacity of coal units is estimated to be 1,008 GW by the end of 2018. Our data covers more than 95% of the total capacity list by CEC.
Sampling strategy	Our analysis does not involve sampling. We used the entire population of existing coal-fired power plants in China from the Global Coal Plant Tracker dataset (Jan 2019).
Data collection	We used an existing dataset with independent modification and updates based on primary research.
Timing and spatial scale	Our data covers operating coal-fired power plants in China up to May 2019, when we stopped data collection and verification and started performing data analysis.
Data exclusions	No data is excluded from the analysis.
Reproducibility	Our analysis does not involve experiment, therefore results can be reproduced using the same data and methodology.
Randomization	Our analysis covers the entire population and does not apply randomization.
Blinding	Our analysis covers the entire population and does not apply blinding.

Did the study involve field work? ☐ Yes ☒ No

Reporting for specific materials, systems and methods

We require information from authors about some types of materials, experimental systems and methods used in many studies. Here, indicate whether each material, system or method listed is relevant to your study. If you are not sure if a list item applies to your research, read the appropriate section before selecting a response.

Materials & experimental systems

n/a	Involved in the study
☒	☐ Antibodies
☒	☐ Eukaryotic cell lines
☒	☐ Palaeontology and archaeology
☒	☐ Animals and other organisms
☒	☐ Human research participants
☒	☐ Clinical data
☒	☐ Dual use research of concern

Methods

n/a	Involved in the study
☒	☐ ChIP-seq
☒	☐ Flow cytometry
☒	☐ MRI-based neuroimaging
